# Input associativity underlies fear memory renewal

**DOI:** 10.1093/nsr/nwab004

**Published:** 2021-01-08

**Authors:** Wei-Guang Li, Yan-Jiao Wu, Xue Gu, Hui-Ran Fan, Qi Wang, Jia-Jie Zhu, Xin Yi, Qin Wang, Qin Jiang, Ying Li, Ti-Fei Yuan, Han Xu, Jiangteng Lu, Nan-Jie Xu, Michael Xi Zhu, Tian-Le Xu

**Affiliations:** Center for Brain Science of Shanghai Children's Medical Center, Shanghai Jiao Tong University School of Medicine, Shanghai 200127, China; Department of Anatomy and Physiology, Shanghai Jiao Tong University School of Medicine, Shanghai 20025, China; Center for Brain Science of Shanghai Children's Medical Center, Shanghai Jiao Tong University School of Medicine, Shanghai 200127, China; Department of Anatomy and Physiology, Shanghai Jiao Tong University School of Medicine, Shanghai 20025, China; Center for Brain Science of Shanghai Children's Medical Center, Shanghai Jiao Tong University School of Medicine, Shanghai 200127, China; Department of Anatomy and Physiology, Shanghai Jiao Tong University School of Medicine, Shanghai 20025, China; Department of Anatomy and Physiology, Shanghai Jiao Tong University School of Medicine, Shanghai 20025, China; Center for Brain Science of Shanghai Children's Medical Center, Shanghai Jiao Tong University School of Medicine, Shanghai 200127, China; Department of Anatomy and Physiology, Shanghai Jiao Tong University School of Medicine, Shanghai 20025, China; Department of Anatomy and Physiology, Shanghai Jiao Tong University School of Medicine, Shanghai 20025, China; Center for Brain Science of Shanghai Children's Medical Center, Shanghai Jiao Tong University School of Medicine, Shanghai 200127, China; Department of Anatomy and Physiology, Shanghai Jiao Tong University School of Medicine, Shanghai 20025, China; Department of Anatomy and Physiology, Shanghai Jiao Tong University School of Medicine, Shanghai 20025, China; Center for Brain Science of Shanghai Children's Medical Center, Shanghai Jiao Tong University School of Medicine, Shanghai 200127, China; Department of Anatomy and Physiology, Shanghai Jiao Tong University School of Medicine, Shanghai 20025, China; Center for Brain Science of Shanghai Children's Medical Center, Shanghai Jiao Tong University School of Medicine, Shanghai 200127, China; Department of Anatomy and Physiology, Shanghai Jiao Tong University School of Medicine, Shanghai 20025, China; Shanghai Key Laboratory of Psychotic Disorders, Shanghai Mental Health Center, Shanghai Jiao Tong University School of Medicine, Shanghai 201108, China; Center for Neuroscience and Department of Neurology of the Second Affiliated Hospital, NHC and CAMS Key Laboratory of Medical Neurobiology, Zhejiang University School of Medicine, Hangzhou 310058, China; Center for Brain Science of Shanghai Children's Medical Center, Shanghai Jiao Tong University School of Medicine, Shanghai 200127, China; Department of Anatomy and Physiology, Shanghai Jiao Tong University School of Medicine, Shanghai 20025, China; Department of Anatomy and Physiology, Shanghai Jiao Tong University School of Medicine, Shanghai 20025, China; Department of Integrative Biology and Pharmacology, McGovern Medical School, University of Texas Health Science Center at Houston, Houston, TX 77030, USA; Center for Brain Science of Shanghai Children's Medical Center, Shanghai Jiao Tong University School of Medicine, Shanghai 200127, China; Department of Anatomy and Physiology, Shanghai Jiao Tong University School of Medicine, Shanghai 20025, China; Shanghai Research Center for Brain Science and Brain-Inspired Intelligence, Shanghai 201210, China

**Keywords:** fear renewal, memory ensembles, input associativity, long-term potentiation, ventral hippocampus, auditory cortex, lateral amygdala

## Abstract

Synaptic associativity, a feature of Hebbian plasticity wherein coactivation of two inputs onto the same neuron produces synergistic actions on postsynaptic activity, is a primary cellular correlate of associative learning. However, whether and how synaptic associativity are implemented into context-dependent relapse of extinguished memory (i.e. fear renewal) is unknown. Here, using an auditory fear conditioning paradigm in mice, we show that fear renewal is determined by the associativity between convergent inputs from the auditory cortex (ACx) and ventral hippocampus (vHPC) onto the lateral amygdala (LA) that reactivate ensembles engaged during learning. Fear renewal enhances synaptic strengths of both ACx to LA and the previously unknown vHPC to LA monosynaptic inputs. While inactivating either of the afferents abolishes fear renewal, optogenetic activation of their input associativity in the LA recapitulates fear renewal. Thus, input associativity underlies fear memory renewal.

## INTRODUCTION

As the foundation of adaptive behaviors, memories are encoded as enduring physical changes of engrams at multiple levels across various brain regions [1,2]. Memories related to traumatic experience often last for a lifetime [3]. Although they can be treated with extinction learning to dampen their expression, the original memory engrams are merely silenced not erased [4–6], and under certain conditions they are reactivated to cause relapse [7]. Typically, in auditory fear conditioning [8–13], a tone as the conditioned stimulus (CS) paired with an aversive foot shock as the unconditioned stimulus (US) produces a long-lasting fear memory. Of note, although the memory of initial CS-US association can be retrieved in a context-independent manner [14,15], extinction (or lack of expression) of such a memory is highly context-dependent [10,16–18]. Thus, fear extinction to the conditioned tone occurs only when the subject is in the same context as where the extinction training was performed, suggesting that the fear memory trace still exists but is kept in a silent state. The extinguished fear can be rapidly retrieved or renewed when the subject is tested with the tone in any context outside where the extinction training was performed, a process referred to as fear renewal [7,10,15,18]. Therefore, context can act as an ‘occasion setter’ that modulates the retrieval of discrete CS-US associations [19]. More specially for fear renewal, both appropriate contexts and the conditioned tone are required but neither alone is sufficient to cause the fear response. This raises the question of how the tone and context-dependent occasion setting work together at the cellular and synaptic levels to mediate the relapse of fear.

In principle, the retrieval of extinguished memory can be viewed as a process that reawakens an engram out of its latent state into one of manifested activity. However, the cellular mechanisms and substrates underlying transformation of an extinguished memory to its renewal remain unclear. Memory-based adaptive behaviors are thought to be a macroscopic manifestation of microscopic plastic changes in synaptic strength such as long-term potentiation (LTP). For fear memory, synaptic plasticity of lateral amygdala (LA) neurons is essential for cued fear learning and this represents a candidate mechanism through which subsets of neurons (i.e. engram) are recruited during fear learning and memory retrieval [20,21].

Combining auditory fear conditioning acquisition/testing, memory engram cell labeling, optogenetics, chemogenetics, anterograde/retrograde tract tracing, intersectional genetics, and *ex vivo* patch-clamp electrophysiology approaches, here we investigate the contributions of the LA-innervating cortical and hippocampal afferents to the expression of fear renewal. We demonstrate that input associativity of convergent tone-associated and context-dependent signaling from the auditory cortex (ACx) and ventral hippocampus (vHPC), respectively, confer fear renewal in an environment distinct from where the earlier fear extinction was established. We

propose that hippocampal and cortical inputs into LA work in a highly associative manner to encode long-term synaptic plasticity necessary for fear renewal, and that the dynamic interplay of two distinct inputs underlies whether or not context-dependent relapse of extinguished fear memory takes place.

## RESULTS

### Fear renewal reactivates memory engram established during fear learning

**Figure 1.**
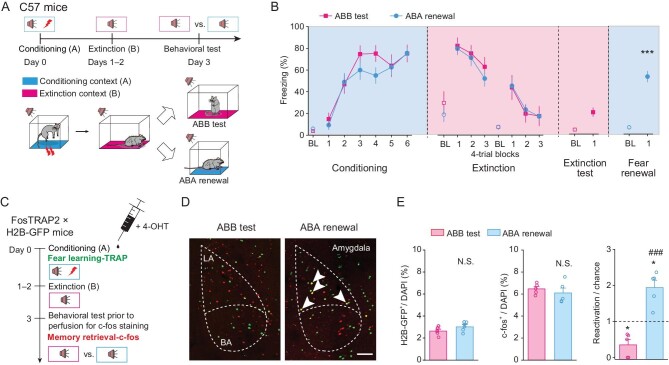
Fear renewal reactivates LA ensembles engaged during fear learning. (A) Schematic representation of the behavioral protocol for mice subject to fear conditioning (day 0, context A), extinction learning (days 1–2, context B), and subsequent extinction test (day 3, context B; referred to as ABB extinction test group thereafter) or fear renewal (day 3, context A; referred to as ABA renewal group thereafter) before slices were prepared for studying synaptic and cellular adaptations *ex vivo*. (B) Freezing responses to the context only (baseline, BL) or CS. While the freezing response during conditioning was calculated by percent freezing time to CS in individual trials, those during extinction learning and fear renewal were calculated by the average freezing responses of four consecutive trials. The same convention was used for the calculation of all behavioral results. Statistics are as follows: two-way repeated measures ANOVA, main effect of behavior, conditioning, F_1,136_ = 2.218, *P* = 0.139; extinction learning, F_1,106_ = 0.232, *P* = 0.631. ^***^P < 0.001 *vs*. extinction test, unpaired Student's *t*-test. ABB test, *n* = 10, ABA renewal, *n* = 13. (C) Schematic representation of the behavioral protocol used before slices were prepared for studying reactivation of fear learning-tagged neurons *ex vivo*. Administration of 4-OHT to TRAP2 (Fos^2A-iCreER^)::H2B-GFP^flox^ (lox-stop-lox-H2B-GFP) mice activates permanent expression of H2B-GFP in neurons active around the time of the injection (i.e. fear learning). (D) Representative image of GFP^+^ (*green*) and c-fos^+^ (*red*) immunofluorescence in the amygdala. The white arrowheads denote co-labeled H2B-GFP^+^/c-fos^+^ cells. Scale bar, 200 μm. (E) Histograms of mean ± S.E.M. with circles denoting individual mice. *Left*, The ABB test and ABA renewal groups displayed similar percentages of GFP^+^ cells among DAPI^+^ cells. *Middle*, ABB test and ABA renewal activated similar percentages of c-fos^+^ cells among DAPI^+^ cells. *Right*, The number of reactivated (GFP^+^/c-fos^+^) cells was significantly higher in the ABA renewal group than in the ABB test group. ABB test, *n* = 5 mice; ABA renewal, *n* = 5 mice. ^*^P < 0.05, compared with the value of 1 (*dashed line*), paired Student's *t*-test. N.S., no significant difference, ^###^*P* < 0.001, ABB test *vs.* ABA renewal, unpaired Student's *t*-test.

A cued fear conditioning paradigm in laboratory rodents, wherein a conditioned stimulus (CS) such as a sound is paired with an aversive unconditioned stimulus (US) such as a foot shock, can produce a lifelong fear memory (Fig. 1A). Repetitive presentation of the tone (CS) alone leads to fear extinction, as seen by the lack of freezing in the extinction context (e.g. magenta floor box, referred to as context B and the test as ABB test) (Fig. 1A and B). However, the fear memory trace is retained and can be rapidly reactivated when the subject is tested with the tone in the conditioning context (e.g. cyan or context A, with the test referred to as ABA renewal) (Fig. 1A and B), or any other context distinct from the extinction context [7].

To understand the cellular mechanisms of extinction and renewal of cued fear, we first asked whether fear renewal reactivates the memory engram previously established during fear learning. We employed the FosTRAP strategy [22–24] to genetically label LA neurons that were activated during fear conditioning. The TRAP (targeted recombination in active populations) system uses the *c-fos* gene locus to drive the expression of tamoxifen-inducible Cre recombinase (CreER), along with a transgenic or virally delivered Cre-dependent effector [25]. When a neuron is active in the presence of tamoxifen or 4-hydroxytamoxifen (4-OHT), CreER enters the nucleus to catalyze recombination, resulting in permanent expression of the effector. In TRAP2::H2B-GFP^flox^ (lox-stop-lox-H2B-GFP) double transgenic mice, injection of 4-OHT 30 min before fear conditioning led to the expression of nuclear histone H2B-conjugated GFP (H2B-GFP) in neurons activated during fear learning. The mice were then subject to extinction training for two days and then tested for extinction or fear renewal on day 3 (Fig. 1C). At 90 min after the test, the animal was sacrificed and the brain processed for immunohistochemistry to detect the expression of endogenous *c-fos* as well as the expression of the H2B-GFP. The extinction test and fear renewal groups displayed similar fractions of H2B-GFP^+^ or c-fos^+^ cells in the LA among 4,6-diamidino-2-phenylindole (DAPI)^+^ cells (Fig. 1D and E), suggesting that similar number of LA neurons are engaged in the fear renewal and extinction groups. However, assessing the reactivation of H2B-GFP^+^ cells via dividing the percentage of the H2B-GFP^+^/c-fos^+^ co-labeled cells among DAPI^+^ cells by the chance percentage [(H2B-GFP^+^/DAPI^+^) × (c-fos^+^/DAPI^+^)] × 100%] [24,26,27] revealed a markedly higher proportion in the fear renewal group than in the extinction test group (Fig. 1D and E). Specifically, we found that fear renewal reactivated around twice the number of learning-engaged LA cells compared with that happening by chance, whereas the extinction test reduced the number by more than half (Fig. 1D and E). These results are consistent with the notion that the fear memory engram is reactivated during renewal, but inhibited by extinction [4,5,7].

### Critical engagement of LA in fear renewal

To directly examine the LA-related neural circuits (Supplementary Fig. 1A) in suppression and renewal of fear responses after extinction [10], we measured neuronal activity of the LA in acute brain slices prepared from mice that were exposed to either extinction test or fear renewal. We found that fear renewal resulted in a marked increase in firing rate and excitability compared with extinction (Supplementary Fig. 1B and C), which were accompanied with a significant decrease in the rheobase but no change in the amplitude of the action potential (AP) (Supplementary Fig. 1D). These results reinforce the importance of LA neuron hyperactivity in fear renewal [28].

To examine whether LA activity is causally linked with the renewal or suppression of cued fear memory, we employed optogenetics to inhibit or activate the LA neurons [29] during the memory test. We first injected an adeno-associated virus (AAV) coding for ArchaerhodopsinT (ArchT), along with GFP for visualization, driven by the Ca^2+^/calmodulin-dependent protein kinase type II subunit α (CaMKIIα) promoter for projection neuron-specific expression (AAV-CaMKIIα-ArchT-GFP) into the LA for neuronal silencing by illuminating the cell bodies (Supplementary Fig. 1E and F). Electrophysiologically, activation of ArchT in LA neurons in slices using 532 nm light evoked an outward current, induced membrane hyperpolarization, and completely suppressed electrical depolarization-induced firing (Supplementary Fig. 1G). Behaviorally, illumination of 532 nm light to inhibit the excitation of LA specifically during the tone exposure in the renewal context attenuated fear renewal (Supplementary Fig. 1H), indicating that activation of LA neurons is necessary for the retrieval of cued fear.

We then injected an AAV expressing ChR2-E123T/T159C (referred to as ChR2), along with mCherry for visualization, driven by the CaMKIIα promoter (AAV-CaMKIIα-ChR2-mCherry) into the LA for neuronal excitation by illuminating the cell bodies (Supplementary Fig. 1I). Illumination of the LA in slices with blue light (473 nm) resulted in inward currents in transduced LA neurons under voltage-clamp at –70 mV, with the amplitude increasing as the light intensity raised (Supplementary Fig. 1J and K). In the current-clamp mode, trains of short blue light pulses (1-ms duration) induced AP firing in ChR2-expressing neurons, with the frequency precisely following that of the photostimulation (Supplementary Fig. 1L and M). Behaviorally, illumination of 473 nm light to excite the LA specifically during tone exposure in extinction context led to an enhanced freezing response (Supplementary Fig. 1N), indicating that increasing LA neuron excitability facilitates fear renewal in conditions that otherwise express extinction. Together, these results demonstrate the necessity and sufficiency of LA neuron excitation in the retrieval of extinguished fear.

We next assessed the influence of synaptic inputs over intrinsic membrane excitability on the renewal-related enhancement of LA neuron excitation. Pharmacological blockade (Supplementary Fig. 2A) of glutamatergic *N*-methyl-D-aspartate receptors (NMDARs) and α-amino-3-hydroxy-5-methyl-4-isoxazole propionate receptors (AMPARs) with CNQX plus D-APV (Supplementary Fig. 2B and C), but not that of γ-aminobutyric acid type A receptors (GABA_A_Rs) with picrotoxin (Supplementary Fig. 2D and E), reversed the increase in firing rate in the fear renewal group. In the presence of CNQX plus D-APV and picrotoxin, the difference in firing rates vanished between the extinction test and fear renewal animals (Supplementary Fig. 2F and G), implying a negligible impact of intrinsic membrane excitability of LA neurons on fear renewal behaviors. Consistently, the frequency, but not amplitude, of spontaneous excitatory postsynaptic currents (sEPSCs), as a proxy of overall synaptic drive in LA neurons, was significantly increased after fear renewal compared with extinction test (Supplementary Fig. 2H–J), implicating a specific enhancement in presynaptic locus. Likewise, fear renewal was attenuated by inhibition of AMPARs and NMDARs with CNQX plus D-APV (Supplementary Fig. 3A–D), but not inhibition of GABA_A_Rs with picrotoxin (Supplementary Fig. 3E–G), at the LA neurons, indicating that excitatory synaptic drive onto LA neurons controls fear renewal.

To distinguish whether fear renewal is selective for the specific CS tone (auditory stimulus) or a generalized response to just loud noise, we subjected the animals that had undergone fear learning and extinction to an unconditioned tone (CS–) that differed from the conditioning tone (CS+). In both the extinction context (context B) and the conditioning context (context A), CS– evoked low freezing responses similar to that elicited by CS+ in the extinction context (Supplementary Fig. 4), indicating that the renewal, which only manifested in context A with CS+, is selective for the particular fear memory acquired during fear learning rather than a generalized fear to sound.

### Characterization of hippocampal and cortical synaptic inputs into amygdala

**Figure 2.**
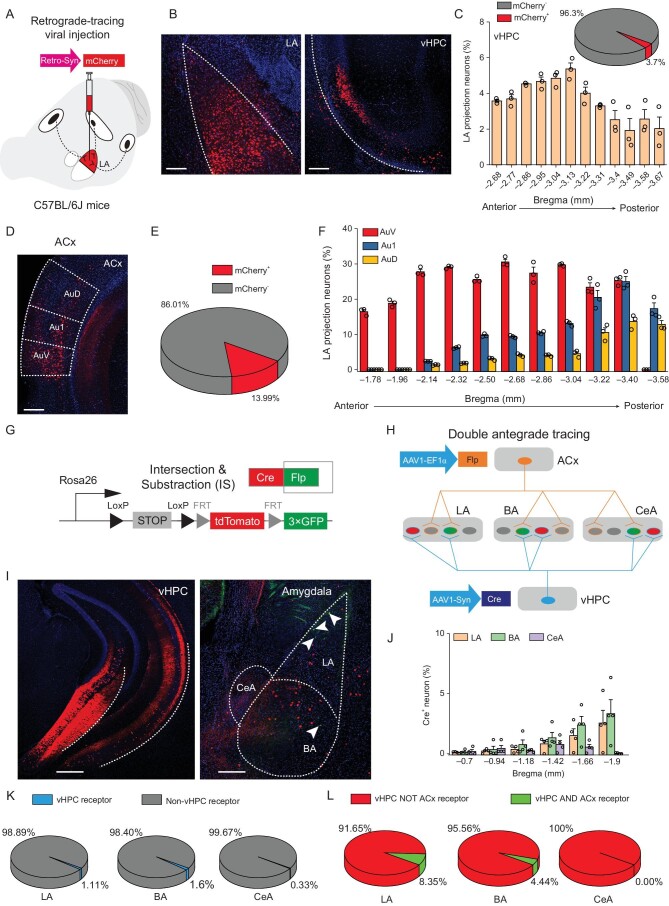
Characterization of hippocampal and cortical synaptic inputs into amygdala. (A–F) Characterization of LA-projecting ACx and vHPC neurons. (A) Schematics of AAV injections to identify LA-projecting neurons in upstream brain regions. (B) Representative image of mCherry expression (*red*) in a mouse that received AAV-retro-Syn-mCherry injection into the LA (*left*), showing a histological example of LA-projecting vHPC neurons (*red*, mCherry-positive). DAPI (*blue*) was used to label nuclei. *Left*, LA, scale bar, 200 μm; *Right*, vHPC, scale bar, 500 μm. (C) Quantification of LA-projecting vHPC neurons. *n* = 3 mice. (D) Representative image of mCherry expression (*red*) showing a histological example of LA-projecting ACx neurons (*red*, mCherry-positive). DAPI (*blue*) was used to label nuclei. Scale bar, 200 μm. (E and F) Quantification of LA-projecting ACx neurons. *n* = 3 mice. (G–L) Anterograde transsynaptic mapping of amygdalar neurons receiving projections from both ACx and vHPC. (G) Schematics of intersection-subtraction (IS) reporter mouse line for genetic targeting of different populations of amygdalar neurons. (H) Schematics of AAV injections to identify amygdalar neurons receiving projections from vHPC and ACx. (I) Representative images of tdTomato (*red*) and GFP (*green*) expression in the vHPC (*left*) and amygdala (*right*). DAPI (*blue*) was used to label nuclei. The white arrowheads denote green cells, which are vHPC- and ACx-innervated, resulting from Cre- plus Flp-driven GFP expression in postsynaptic neuronal targets. *Left*, vHPC, scale bar, 500 μm; *Right*, amygdala, scale bar, 200 μm. (J–L) Quantification of vHPC receptors (*red* cells plus *green* cells, J and K) and vHPC plus ACx receptors (*green*, L) among the vHPC receptors (*red* plus *green*, L) in the LA, BA, and CeA. *n* = 4 mice.

Given that fear renewal requires both proper context and conditioned cue, synaptic inputs encoding context and sensory information may converge onto amygdala neurons to drive the fear response. To identify pathways that project to LA neurons and specifically associate auditory stimulus with context to drive fear renewal, we first performed retrograde tracing of long-range inputs by injecting the LA with a retrograde AAV that expresses the fluorescent reporter, mCherry, under the control of the human synapsin promoter (rAAV2-retro-Syn-mCherry) (Fig. 2A). Consistent with the role of the hippocampus in processing contextual information in fear renewal [15,30–35], many retrogradely labeled LA-projecting neurons were found in the ventral hippocampus (vHPC, Fig. 2B and C). In addition, labeled neurons were also found in other brain areas, including the auditory cortex (ACx) (Fig. 2D–F), but much less in the dorsal hippocampus (dHPC) (Supplementary Fig. 5).

To visualize the hippocampal and cortical inputs into the amygdala and their convergence, we then performed AAV-mediated anterograde transsynaptic tracing [36] using the high-performance intersection-subtraction (IS) reporter mice, in which the fluorescent protein tdTomato is expressed in the presence of Cre, whereas another fluorescent protein GFP is expressed when both Cre and Flp are present [37] (Fig. 2G). AAV1-Cre, an anterograde transsynaptic-spread viral serotyxpe, was transduced into presynaptic ACx neurons in the IS mice, which enabled effective and unbiased expression of the Cre-driven tdTomato in postsynaptic targets (Supplementary Fig. 6A). We found that ACx neurons projected preferentially to the LA, moderately to the BA, and minimally to the CeA (Supplementary Fig. 6B–D). In another set of experiments, AAV1-Cre and AAV1-Flp were transduced into presynaptic vHPC and ACx, respectively, of the IS mice. In these mice, neurons innervated by only the vHPC were tagged with tdTomato, while neurons innervated by both the vHPC and the ACx were tagged with GFP (Fig. 2H). The proportions of neurons in the LA, BA, and CeA that received vHPC projections (i.e. red cells plus green cells) were all low (0.33–1.6%), but among them more LA neurons (8.35%) received projections from both vHPC and ACx than BA neurons (4.44%) and no CeA neurons received convergent inputs from both brain areas (Fig. 2I–L). Together with the intersectional data on ACx projecting neurons, our results demonstrate that LA neurons more likely receive convergent inputs from auditory and contextual-representation neurons than other areas of the amygdala, which may underlie the mechanism of fear renewal.

### Fear renewal recruits presynaptic associativity of convergent cortical and hippocampal inputs into LA

**Figure 3.**
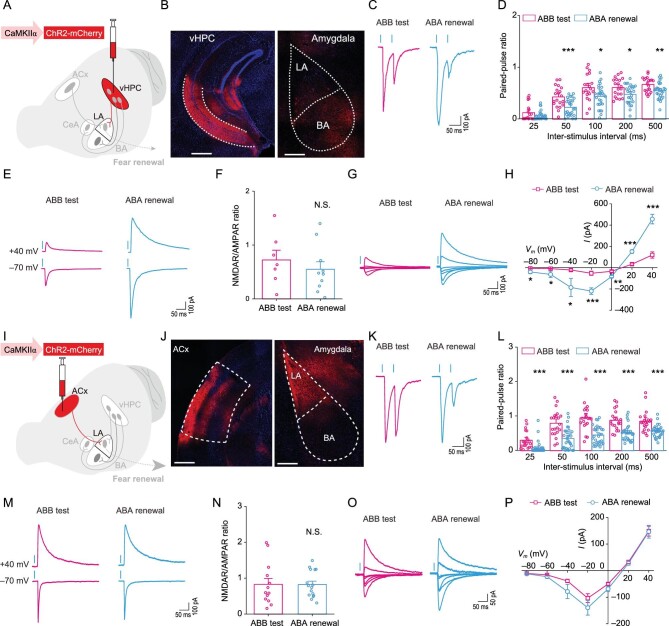
Fear renewal enhances convergent hippocampal and cortical inputs into LA. (A–D and I–L) Effects of fear renewal on paired-pulse ratios (PPRs) for oEPSCs at the vHPC → LA (A–D) and ACx → LA (I–L) projections. (A and I) Schematics of AAV injections. (B and J) Representative images of mCherry expression in injected areas and in amygdala. Scale bars: *Left* 500 μm, *Right* 200 μm. (C and K) Representative traces of oEPSCs at the vHPC → LA (C) or ACx → LA (K) synapses in the extinction test and fear renewal groups induced by paired photostimulations (blue vertical bars) with a 50-ms interval. (D and L) Histograms of mean ± S.E.M. with circles denoting individual neurons. (D) vHPC → LA. Statistics: two-way repeated measures ANOVA, main effect of behavior, F_1,238_ = 34.790, *P* < 0.001; ^*^P < 0.05, ^**^P < 0.01, ^***^P < 0.001, unpaired Student's *t*-test. ABB test, *n* = 20 neurons from six mice; ABA renewal, *n* = 28 neurons from seven mice. (L) ACx → LA. Statistics: two-way repeated measures ANOVA, main effect of behavior, F_1,258_ = 110.093, *P* < 0.001; ^***^P < 0.001, unpaired Student's *t*-test; ABB test, *n* = 21 neurons from four mice; ABA renewal, *n* = 31 neurons from five mice. (E–H and M–P) Effects of fear renewal on NMDAR/AMPAR ratios (E, F, M, and N) and current-voltage relationships of NMDAR-mediated synaptic currents (G, H, O, and P) at the vHPC → LA (E–H) or ACx → LA (M–P) projections. (E and M) Representative traces of AMPAR- and NMDAR-mediated currents in response to photostimulation of vHPC fibers at the vHPC → LA (E) or ACx fibers at the ACx → LA (M) synapses in extinction test and fear renewal groups. (F and N) Ratios of peak NMDAR- to AMPAR-mediated currents at the vHPC → LA (F) and ACx → LA (N) synapses for individual neurons and their summaries. (F) Statistics: N.S., no significant difference, unpaired Student's *t*-test. ABB test, *n* = 10 neurons from four mice; ABA renewal, *n* = 7 neurons from two mice. (N) Statistics: N.S., no significant difference, unpaired Student's *t*-test. ABB test, *n* = 13 neurons from five mice; ABA renewal, *n* = 16 neurons from four mice. (G and O) Representative traces showing current-voltage (I-V) relationships of NMDAR-mediated synaptic currents at the vHPC → LA (G) and ACx → LA (O) projections. (H) Summary I-V relationship at vHPC → LA. Statistics: two-way repeated measures ANOVA, main effect of behavior, F_1,110_ = 0.017, *P* = 0.897; ^*^P < 0.05, ^**^P < 0.01, ^***^P < 0.001, unpaired Student's *t*-test. ABB test, *n* = 10 neurons from four mice; ABA renewal, *n* = 6 neurons from two mice. (P) Summary I-V relationship at ACx → LA. Statistics: two-way repeated measures ANOVA, main effect of behavior, F_1,243_ = 3.944, *P* = 0.048. ABB test, *n* = 17 neurons from six mice; ABA renewal, *n* = 18 neurons from six mice.

To explore functional connectivity of vHPC or ACx to LA neurons, we recorded optogenetically activated synaptic responses of LA neurons in acute brain slices from mice subject to extinction test or fear renewal. To activate the vHPC pathway, we injected AAV-CaMKIIα-ChR2-mCherry to vHPC of wild type C57BL/6J mice for projection neuron-specific expression and optogenetic stimulation of targeted terminals in the LA (Fig. 3A and B). Prominent optically evoked excitatory postsynaptic currents (oEPSCs) were detected in every recorded LA cell voltage clamped at –70 mV in the brain slice (Supplementary Fig. 7A–C), which were blocked by the voltage-gated sodium channel antagonist, tetrodotoxin (TTX), but appeared again with the addition of potassium channel antagonist, 4-AP, demonstrating monosynaptic inputs [38] from vHPC to LA (Supplementary Fig. 7B and C). As a control, light delivery to the brain slices from animals with injection of AAV-CaMKIIα-mCherry control virus to vHPC did not evoke oEPSCs in the recorded LA cells (data not shown). To examine whether fear renewal is associated with adaptive changes of this vHPC → LA synaptic connectivity, the mice were subject to either extinction test or fear renewal before *ex vivo* whole-cell recordings from LA neurons in brain slices were made. With paired light pulses to stimulate vHPC afferents at varying interstimulus intervals, we measured the paired-pulse ratios (PPRs) for oEPSCs, which are inversely related to the presynaptic release probability [39]. The PPRs were significantly decreased in the fear renewal group compared with those in the

extinction test group (Fig. 3C and D), suggesting that fear renewal enhances the presynaptic release probability at vHPC → LA synapses. By holding the patched cell at either +40 or −70 mV, we assessed oEPSCs mediated by NMDARs and AMPARs, respectively, and observed prominent increases in the amplitudes of both NMDAR and AMPAR oEPSCs in the fear renewal group compared with the extinction test group, without a significant change in the NMDAR/AMPAR ratio (Fig. 3E and F). In addition, plotting the NMDAR current amplitudes against different voltages revealed that fear renewal was associated with a significant enhancement of NMDAR-mediated synaptic responses at vHPC → LA synapses (Fig. 3G and H).

It has been proposed that fear memory engram also involves modifications of synaptic strengths of the cued auditory inputs into the amygdala [8,10,20,40]. Thus, we next examined plasticity changes of ACx → LA projections associated with fear renewal. From mice that received the injection of AAV-CaMKIIα-ChR2-mCherry (Fig. 3I and J) but not that of AAV-CaMKIIα-mCherry (data not shown) into ACx and subject to either extinction test or fear renewal, *ex vivo* whole-cell recordings of LA neurons combined with light-induced optogenetic stimulation of ACx fibers were performed. As with the vHPC → LA pathway, the PPRs for oEPSCs of the ACx → LA inputs, which represented monosynaptic synapses (Supplementary Fig. 7D−F), were significantly decreased in the fear renewal group compared with the extinction test group (Fig. 3K and L), indicating also an enhancement of the presynaptic release probability in the ACx → LA pathway in fear renewal. However, unlike the vHPC → LA pathway, no significant difference in the NMDAR and AMPAR oEPSC amplitudes or their ratio was found between the fear renewal and extinction test groups for the ACx → LA synapses (Fig. 3M and N). Plotting the NMDAR current amplitudes against different voltages revealed a marginally significant change (two-way repeated-measures ANOVA, main effect of group, F_1,243_ = 3.944, *P* = 0.048) of NMDAR-mediated synaptic responses at ACx → LA synapses between the two groups (Fig. 3O and P). As a negative control, no significant changes in PPRs were detected in the somatosensory cortex (SCx) → LA projections (Supplementary Fig. 8A–D), even toward the same postsynaptic cells as those targeted by ACx (Supplementary Fig. 8E–M), as demonstrated with a dual optogenetic method that permits independent optical excitation of distinct neural populations [41]. Thus, fear renewal recruits a presynaptic associativity of two convergent pathways of cue-related and context-dependent transmissions from ACx and vHPC, respectively, but not that from SCx, to LA.

### Fear renewal requires synaptic associativity of convergent cortical and hippocampal inputs to LA

**Figure 4.**
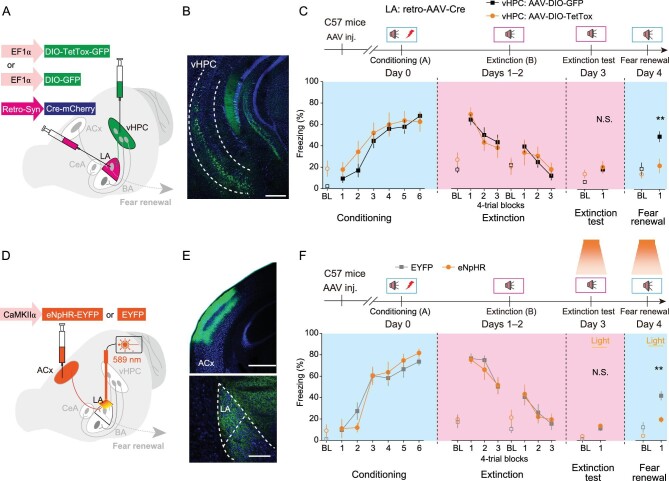
Fear renewal requires both cortical and hippocampal inputs into LA. (A–C) Effects of silencing vHPC → LA projection on fear learning, extinction learning, extinction test, and fear renewal. (A) Experimental schemes. (B) Representative image of GFP expression (*green*) in a mouse that received both retro-AAV-Syn-Cre-mCherry injection into the LA and AAV-DIO-GFP injection into the vHPC. DAPI (*blue*) was used to label nuclei. Scale bar, 500 μm. (C) *Upper*, behavioral protocols. *Lower*, time course of freezing responses to the context only (baseline, BL) or CS. Statistics are as follows: two-way repeated measures ANOVA, main effect of AAV, conditioning, F_1,148_ = 2.188, *P* = 0.141; extinction learning, F_1,148_ = 0.037, *P* = 0.848; N.S., no significant difference, ^**^P < 0.01, unpaired Student's *t*-test. GFP, *n* = 16, TetTox, *n* = 9. (D–F) Effects of optogenetic silencing of ACx → LA projection on fear renewal. (D) Experimental schemes. (E) Representative images of eNpHR-EYFP expression (*green*) in a mouse that received AAV-CaMKIIα-eNpHR-EYFP into ACx. *Upper*, ACx, scale bar, 500 μm; *Lower*, LA, scale bar, 200 μm. DAPI (*blue*) was used to label nuclei. (F) *Upper*, behavioral protocols. *Lower*, time course of freezing response to the context only (baseline, BL) or CS. Statistics are as follows: two-way repeated measures ANOVA, main effect of AAV, conditioning, F_1,118_ = 0.092, *P* = 0.763; extinction learning, F_1,118_ = 0.066, *P* = 0.798; N.S., no significant difference, ^**^P < 0.01, unpaired Student's *t*-test. EYFP, *n* = 10, eNpHR, *n* = 10.

We then asked whether these convergent synaptic inputs to LA are required for fear renewal. Mice were injected in the LA with retrograde retro-AAV-Syn-Cre-mCherry, followed by an injection into the vHPC of an AAV that expresses, in a Cre-dependent manner (double-floxed inverted orientated, DIO), the light chain of tetanus toxin (TetTox), along with GFP for visualization (AAV-DIO-TetTox-GFP). This allows specific inactivation of synaptic transmission in vHPC neurons that project to the LA. AAV-DIO-GFP, which expresses GFP only, was used as the control (Fig. 4A and B). We found that inactivation of vHPC neurons projecting to LA by targeted expression of TetTox markedly reduced freezing time during fear renewal, without an effect on fear conditioning, extinction learning, or extinction test (Fig. 4C).

We then examined the causality of synaptic potentiation of ACx → LA projections in fear renewal by performing a pathway-specific optogenetic inhibition of the ACx → LA projections during the tone test after extinction. To this end, we injected an AAV coding for enhanced halorhodopsin (eNpHR), along with EYFP for visualization, driven by the CaMKIIα promoter for projection neuron-specific expression (AAV-CaMKIIα-eNpHR-EYFP), into the ACx for neuronal silencing by illuminating the axonal terminals in LA (Fig. 4D and E). Electrophysiologically, exposure of yellow light (589 nm) to inhibit ACx → LA axonal terminals significantly reduced corticoamygdalar transmission by electrical stimulation of ACx (Supplementary Fig. 9). Behaviorally, optogenetic inhibition of ACx → LA projections significantly reduced freezing time during fear renewal (Fig. 4F).

**Figure 5.**
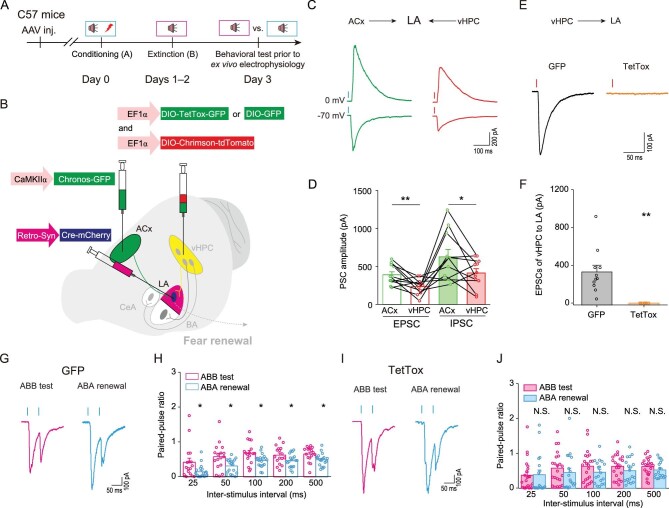
Interdependence of vHPC and ACx inputs to the LA on fear renewal-related synaptic adaptations. Effects of silencing LA-projecting vHPC neurons on synaptic adaptations of ACx → LA projections during fear renewal test. (A) Experimental schemes. (B) Schematics of AAV injections. (C and D) Comparison of the oEPSCs and oIPSCs at ACx → LA and vHPC → LA projections within the same neurons. (C) Representative traces of oEPSCs (holding voltage = –70 mV) or oIPSCs (holding voltage = 0 mV) at ACx → LA and vHPC → LA projections recorded in the same neurons by photostimulations (vertical bars) of blue- and red-light-sensitive Chronos-GFP and Chrimson-tdTomato fibers, respectively. The vertical bar above each trace indicates the photostimulation of blue (λ = 473 nm) or red (λ = 638 nm) light. (D) Statistical results: ^*^P < 0.05, ^**^P < 0.01, paired Student's *t*-test. *n* = 11 neurons from eight mice. (E and F) Electrophysiological corroboration of silencing of the vHPC → LA projections. (E) Representative traces of oEPSCs evoked by optogenetic stimulation of the vHPC-LA pathway by photostimulation of red-light-sensitive Chrimson-tdTomato in vHPC fibers. The vertical bars above traces indicate the photostimulations of red light (λ = 638 nm). (F) Statistic results: ^**^P < 0.01, unpaired Student's *t*-test. GFP: *n* = 11 neurons from seven mice; TetTox: *n* = 8 neurons from four mice. (G and I) Representative traces of oEPSCs induced by paired photostimulations (blue vertical bars) of the ACx-LA pathway with 50-ms intervals from control (G) and vHPC → LA projection silenced mice (I). (H and J) Histograms of mean ± S.E.M. with circles denoting individual neurons. Statistics are as follows: two-way repeated measures ANOVA, main effect of behavior, (H) GFP, F_1,178_ = 28.249, *P* < 0.001; (J), TetTox, F_1,198_ = 3.110, *P* = 0.079; N.S., no significant difference, ^*^P < 0.05, unpaired Student's *t*-test. (H) GFP, ABB test, *n* = 17 neurons from six mice; ABA renewal, *n* = 19 neurons from seven mice; (J) TetTox, ABB test, *n* = 22 neurons from seven mice; ABA renewal, *n* = 18 neurons from seven mice.

We next investigated synaptic associativity of vHPC → LA and ACx → LA projections in fear renewal. The intersectional viral approaches shown in Fig. 2G–L yielded a rather low percentage of neurons that received convergent inputs from vHPC and ACx, probably because of the low efficiency of labeling. As an alternative, we also assessed the proportion of neurons receiving convergent inputs from vHPC and ACx using the dual optogenetic approach (Supplementary Fig. 10A) that allowed for independent optical excitation of distinct neural populations [41]. Overcoming the limitations of the transsynaptic [36] and intersectional labeling [37], we found that the majority of LA neurons received joint synaptic inputs from both ACx and vHPC, and the proportions of jointly innervated neurons in BA and CeA were lower than in LA (Supplementary Fig. 10), consistent with the finding that most LA neurons are responsive to both auditory CS and shock US [42]. To examine whether the behavioral loss of fear renewal by silencing the synaptic transmission of vHPC neurons to LA was a result of insufficient reactivation of the fear memory engram in the ACx → LA pathway, we used the same approach as that shown in Fig. 4A, with the additional expression of a blue-light-sensitive opsin, Chronos-GFP in ACx, and red-light-sensitive Chrimson-tdTomato in vHPC. This enabled optogenetic stimulations of ACx and vHPC terminals separately in the LA of the same test subject [41] (Fig. 5A and B). In the GFP group, we found both prominent ACx- and vHPC-driven oEPSCs when holding the cell at –70 mV, as well as feedforward optically evoked inhibitory postsynaptic currents (oIPSCs) when holding at 0 mV, in the same LA neurons, using blue and red lights, respectively (Fig. 5C and D). As expected, the expression of TetTox in vHPC neurons projecting to the LA abolished vHPC → LA oEPSCs (Fig. 5E and F), but more intriguingly, this manipulation also diminished the difference between fear renewal and extinction test groups in the synaptic adaptations of the ACx → LA pathway, as revealed by the PPRs in response to paired photostimulation of the ACx inputs (Fig. 5G–J). Thus, the fear renewal-related synaptic potentiation of cortical and hippocampal inputs, as demonstrated above in Fig. 3C, D, K and L, operates in an interdependent manner, and the synaptic associativity between them is necessary for fear renewal.

### Synaptic associativity with fear renewal in LA is abolished by additional extinction in the renewal context

To further address the behavioral consequence of synaptic associativity in LA for fear renewal, we sought to determine whether the fear renewal-related synaptic adaptations in the ACx → LA and vHPC → LA pathways could be reversed by additional extinction. To extinguish the fear response in the renewal context, the test animals were given repeated CS presentations without footshock in the renewal context (i.e. context A), whereas the control animals were kept at the homecage to maintain the fear renewal response (Supplementary Fig. 11). As expected, the control mice maintained the high fear response, while the extinguished animals exhibited a markedly reduced freezing response (Supplementary Fig. 11A and B), indicating that fear renewal, like a fear memory, can be extinguished by the repeated

exposure of CS only in the renewal context. Interestingly, the synaptic adaptions associated with fear renewal in terms of sEPSCs in LA neurons and PPRs of the ACx → LA projections, were largely reversed by the additional extinction training (Supplementary Fig. 11C–E, H and I). However, the PPRs of the vHPC → LA pathway did not reverse (Supplementary Fig. 11F and G). The resistance of fear renewal-induced vHPC → LA projection plasticity to change in response to additional extinction training in the renewal context further indicates the context-related input specificity of the vHPC → LA synapses in fear-memory. These dynamic synaptic adaptions in the LA underlie the opposite behavioral consequences to CS in a context-dependent manner.

### Boosting synaptic associativity in LA drives fear renewal

The fear renewal paradigm involves both contextual information and auditory cues. To determine whether the synaptic associativity of the vHPC and

**Figure 6.**
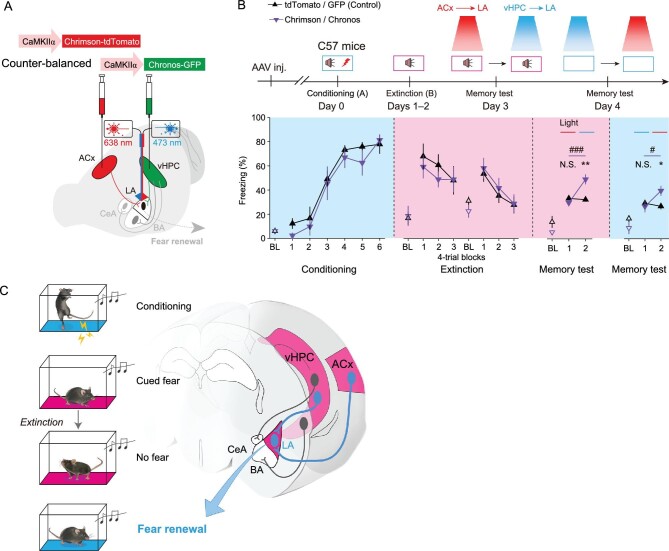
Effects of optogenetic activation of vHPC → LA or ACx → LA projections on fear renewal in the absence of proper context or sensory cues. (A) Experimental schemes for viral injections and optical fiber implantation. One optical fiber was implanted for each animal and the light source (either 638 nm or 473 nm) was switched in between the tests. (B) *Upper*, behavioral protocols. *Lower*, time course of freezing response to the context only (baseline, BL) or CS. While the freezing response during conditioning was calculated by the percent freezing time CS during individual trials, those during extinction learning, extinction test, and memory tests were calculated by the average freezing responses of four consecutive trials. Statistics are as follows: two-way repeated measures ANOVA, main effect of AAV, conditioning, F_1,82_ = 1.960, *P* = 0.166; extinction learning, F_1,82_ = 0.060, *P* = 0.807; N.S., no significant difference, ^*^P < 0.05, ^**^P < 0.01, tdTomato/GFP (Control) *vs*. Chrimson/Chronos, unpaired Student's *t*-test; ^#^*P* < 0.05, ^###^*P* < 0.001, comparison as indicated, paired Student's *t*-test. tdTomato/GFP (Control), *n* = 8, Chrimson/Chronos, *n* = 6. (C) Scheme for synaptic associativity of convergent hippocampal and auditory cortical inputs into the amygdala for dictating fear renewal. Synaptic associativity of convergent cortical and hippocampal inputs, namely cue-associated ACx → LA and context-dependent vHPC → LA pathways, respectively, underlies the reactivation of LA ensembles engaged in fear learning to enable fear renewal. This associativity likely plays an indispensable role in ensuring the specificity of fear renewal with both the proper context and conditioning cue. Please see text for more details.

ACx inputs into the LA is sufficient for fear renewal, we used optogenetic stimulation to substitute the corresponding components during the extinction and renewal tests. To allow selective photostimulation of either vHPC → LA or ACx → LA projections between behavioral tests in the same animal, two light sources were coupled to a single optical fiber implanted into the LA (Fig. 6A). Given that photostimulation of vHPC projections typically does not activate the same ensembles that transmit contextual cues of the extinction, it fulfills the requirement of fear renewal, as a ‘context’ distinct from the extinction context (i.e. context B) [7]. Interestingly, in the extinction context paired with the cued tone, optogenetic stimulation of vHPC → LA, but not ACx → LA, projections elicited increased freezing responses, indicative of fear renewal (Fig. 6B, Memory test day 3). Conversely, in the

renewal context omitting the cued tone, optogenetic stimulation of ACx → LA, but not vHPC → LA, projections triggered fear renewal (Fig. 6B, Memory test day 4). Thus, activation of vHPC → LA or ACx → LA inputs in the absence of the corresponding contextual information or auditory cues, respectively, is sufficient for fear renewal. Together with the data on inhibiting these projections shown above, these results demonstrate both the necessity and sufficiency of synaptic associativity at vHPC and ACx inputs to LA in the retrieval of cued fear memory (Fig. 6C).

## DISCUSSION

Collectively, our present findings delineate an indispensable role of input associativity between two distinct pathways of cortical and hippocampal inputs into LA in the renewal of cued fear memory. The LA is well known to code for many aspects of fear memory, such as longitudinal transformation of long-term associative memory from fear learning to extinction [42], transverse discrimination [43], or interaction [44] of different memories, and affirming the identities of individual memories [45], each with specific synaptic inputs. Our findings here demonstrate that fear renewal utilizes the associativity rule of Hebbian learning and memory [46] by linking presynaptic plasticity of two independent inputs in the LA. This presynaptic associativity of convergent cortical and hippocampal inputs, namely coincident detection of tone-related ACx → LA and context-dependent vHPC → LA pathways, respectively, underlies the reactivation of LA ensembles engaged in fear learning to enable fear renewal (Fig. 6C). This previously unknown form of associativity ensures the specificity and fidelity of fear renewal that requires both the proper context and conditioning cue.

As a primary cellular correlate of learning and memory, LTP of synaptic transmission possesses three key features, namely associativity, specificity, and cooperativity, which fulfil the fundamental rules of the Hebbian learning and memory [46]. Synaptic cooperativity of LTP means the property of nearly simultaneous activation of a large number of afferent axons to induce LTP and implicates that only events which trigger sufficient inputs can result in memory storage [47]. Input specificity of LTP, manifested at active, but not inactive, presynaptic afferents to the postsynaptic cell, ensures the formation of discriminative fear memories [43,45]. Synaptic associativity historically refers to the observation that when weak stimulation of a single pathway is insufficient for the induction of LTP, simultaneous strong stimulation of another pathway will induce LTP at both pathways, which is a primary cellular correlate of associative learning [8,12,48] and is even used to guide the artificial implantation of memories [20,49]. Here, we describe a previously uncharacterized form of synaptic associativity, namely that coactivation of two inputs onto the same neuron produces synergistic actions on postsynaptic activity, and constitutes a neural correlate underlying context-dependent relapse of extinguished memory.

As an ideal experimental model for the recurrent distressing intrusion symptoms of subjects with posttraumatic stress disorder, fear renewal best recapitulates the context-dependent relapse of extinguished traumatic memories [7]. It has long been considered that this contextual control of extinguished fear memory critically depends on the hippocampus, as pharmacological inhibition of either the dorsal [30] or ventral [32] areas of this structure leads to a context-independent expression of extinction and prevents fear renewal. Remarkedly, vHPC sends monosynaptic projections to the central amygdala [15] and a strong feedforward inhibitory circuit to the medial prefrontal cortex [35], both of which are involved in the contextual control of extinguished fear memory. Here, we identified the vHPC → LA projection as a new pathway that regulates fear renewal through association with the ACx → LA pathway. First, by combining retrograde and intersectional anterograde neuronal tracing of the hippocampal-amygdala circuits, we anatomically characterized a previously neglected vHPC → LA projection (Fig. 2). Of note, even though the abundance order of amygdala neuronal subpopulations receiving the vHPC projections was BA > LA > CeA, and that receiving the ACx projections was LA > BA > CeA, LA possessed the largest proportion of neurons that received both vHPC- and ACx-projections, which was followed by BA and CeA, with the latter exhibiting nearly no neurons that received both vHPC- and ACx-projections. This convergence of vHPC and ACx inputs into a subset of LA neurons lays an anatomic foundation for the synaptic association of vHPC → LA with ACx → LA projections. Second, by taking advantage of a dual optogenetic approach that permits independent optical excitation of distinct neural populations, we consistently detected robust ACx- and vHPC-derived oEPSCs in the same postsynaptic cells in LA, but with a much lower probability in BA or CeA (Supplementary Fig. 10), functionally validating that two independent inputs onto the same LA neurons for associative interaction conjointly control the postsynaptic activity (Fig. 5A–C). This functional approach also helped overcome the limitation of the viral tracing method described above, which yielded underestimation of the vHPC- and ACx-innervated neuronal populations because of the low labeling efficiency. Third, as the straightforward evidence for synaptic associativity of convergent ACx → LA and vHPC → LA projections, fear renewal resulted in several cellular consequences including adaptive presynaptic changes at both these projections as reflected by PPRs. Interestingly, we observed no significant postsynaptic alterations in the two projections measured as NMDAR/AMPAR oEPSC ratios (Fig. 3). In addition, the synaptic strength of SCx → LA projection did not differ between the extinction test and fear renewal subjects, implicating a dispensable role of this pathway (Supplementary Fig. 8). Fourth, when the synaptic transmission of vHPC neurons to LA was silenced, the adaptive changes of ACx → LA projection induced by fear renewal also concomitantly diminished (Fig. 5). This dependence of one pathway on the other further indicates synaptic associativity of the convergent ACx → LA and vHPC → LA projections in fear renewal. Fifth, blockade of synaptic transmission of vHPC neurons projecting to the LA selectively abolished fear renewal but did not affect fear conditioning and extinction learning, indicating the behavioral significance of vHPC → LA projection for fear renewal. Likewise, pathway-specific optogenetic inhibition of the ACx → LA projections also significantly reduced freezing during fear renewal (Fig. 4). Sixth, additional extinction training in the renewal context following fear renewal abolished the adaptive changes of ACx → LA, but not that of vHPC → LA, induced by fear renewal (Supplementary Fig. 11). This not only supports the necessity of synaptic associativity in LA for fear renewal, but also suggests input specificity of LA for the complex regulation of memory-related behaviors. Seventh, selective photostimulation of vHPC → LA or ACx → LA projections in the absence of the contextual information or auditory cues, respectively, but not the other way around, successfully reconstructed fear renewal behavior (Fig. 6A and B). This result not only further supports the specific role of these two pathways in coding for contextual or cued signals, but also establishes the sufficiency of synaptic associativity between vHPC → LA and ACx → LA projections in fear renewal. Together, the synaptic associativity that we discover between the two pathways fills a major knowledge gap in understanding how a fear memory transforms from an extinguished state to a renewed state in a context-dependent manner (Fig. 6C).

It may be counter-intuitive that the tone-related ACx → LA pathway undergoes marked pre- but not post-synaptic plasticity changes in animals experiencing fear renewal from extinction. Based on the notion that synaptic projections from ACx to LA are modified during acquisition and expression of fear memory to auditory stimulation [50], our study, by focusing on the context-dependent relapse of extinguished fear memory for the first time, further strengthened the proposed link between synaptic plasticity in auditory inputs to the amygdala and learned fear. For auditory fear conditioning, the LA is a locus of convergence for both auditory (i.e. CS) and somatosensory (i.e. US) information and is a plausible site for CS-US association by recruiting distinct synaptic projections [9]. Consistent with this idea, our results indicate that fear renewal recruits synaptic associativity of convergent ACx and vHPC inputs into LA, which likely underlies the fact that fear renewal requires both a proper context and the conditioned cue. Meanwhile, fear renewal seemed not to affect the strength of somatosensory inputs to the LA (Supplementary Fig. 8). Thus, fear renewal, to some extent, represents a previously undefined ‘learning’ process likely relying on the synaptic associativity of auditory and contextual pathways into LA.

Mechanistically, monitoring and manipulating neuronal activity patterns in the LA, vHPC, and ACx in real time *in vivo* will help to further delineate the associative manner, in which the transformation of memory engrams during fear renewal encodes the cued and contextual representations. Our results in terms of microinfusion of pharmacological agents (Supplementary Fig. 3) clearly implicate that the altered glutamatergic transmission in the LA indeed confers fear renewal [51]. More generally, LA neurons may also integrate additional inputs other than hippocampal and auditory cortical inputs, including those from the medial prefrontal cortex [52] and anterior cingulate cortex [53], more likely in a similar synaptically associative manner to that identified in the present study, to convey more complex information for the expression of an appropriate fear response.

For the roles of vHPC in fear renewal, it has been reported that vHPC neurons send monosynaptic projections to the CeA [15] in addition to a strong feedforward inhibitory circuit to the infralimbic subdivision of mPFC [35], both of which are required for fear renewal. Besides, compared with animals that underwent extinction test, those that experienced fear renewal showed preferential activation of vHPC neurons projecting to both BA and the prelimbic subdivision of mPFC [34]. Here, we identified the vHPC → LA projection to be critical for fear renewal, thus unveiling a previously unknown circuit that may underlie direct reactivation of the extinguished fear memory engram. Interestingly, although the vHPC → LA pathway serves a selective role in fear renewal, it does not encode the cued fear memory in a particular context, meaning that the renewal does not reflect a pure reactivation of the same pathway. Here, at least a part of the vHPC → LA pathway critical for fear renewal did not participate in the encoding process. This raises an interesting question of how contextual information is selectively associated with cued fear memories to enable their selective renewal at a later time. Based on the observation that fear renewal significantly enhanced, but extinction reduced, reactivation of the memory engram established during fear learning (Fig. 1), we suggest that removal of extinction-related inhibition, also known as disinhibition [54], most likely underlies the mechanism of synaptic associativity between convergent vHPC → LA and ACx → LA pathways that drives fear renewal.

Along the transformation axis of fear memory, although fear conditioning acts in a projection-specific manner to induce persistent presynaptic depression of the excitatory sensory inputs to, and thereby reducing the inhibitory outputs of, parvalbumin-positive interneurons in LA [55], fear extinction actually recruits amygdalar GABAergic interneurons to suppress the original fear memory [56,57]. As presynaptic γ-aminobutyric acid type B receptors (GABA_B_Rs) are known to mediate selective suppression of glutamatergic inputs to excitatory neurons in the LA [58], it is expected that fear renewal would recruit a mechanism by which GABA release is modified to alter the activity of presynaptic GABA_B_Rs and in turn allow the excitatory synaptic plasticity associated with fear renewal. Under such a scenario, the gain of synaptic strength at the ACx → LA pathway in fear renewal likely represents removal of a GABA_B_R-mediated inhibition. Future studies should be implemented to help understand not only how the extinction context activates a specific subset of vHPC → LA projections that recruit LA GABAergic interneurons to carry out inhibition, but also how such inhibition is removed in fear renewal through activation of a different set of vHPC → LA projections. As optogenetic stimulation of vHPC → LA projections evoked fear renewal despite the presence of the extinction context (Fig. 6A and B), a lack of the extinction context-related vHPC → LA inputs alone is likely insufficient to cause the removal of the GABA_B_R inhibition. It is plausible that the GABA_B_R inhibition is removed by activation of other vHPC → LA inputs outside those involved in extinction training, regardless of whether or not the extinction context-related inputs are still active. These other vHPC → LA inputs do not need to overlap or be identical to those involved during fear conditioning. Consistent with this interpretation, fear renewal does not always require CS presentation in the conditioning context, as it occurs in any contexts outside where the extinction training was performed [7].

It is important to note that the context-dependent fear renewal is not equivalent to the retrieval of contextual fear memory [15], because after undergoing fear learning and extinction, mice showed very low freezing (baseline freezing) responses when exposed again to the conditioning context without the CS presentation. Thus, the finding that optogenetic stimulation of ACx → LA projections evoked fear renewal in the absence of the cued tone (Fig. 6A and B) not only strengthens the associative specificity of the convergent ACx → LA and vHPC → LA projections in fear renewal, but also implicates the capability of the ACx → LA pathway to modify vHPC → LA inputs to drive fear renewal in the conditioning context and even novel contexts. Overall, the presynaptic associativity between cortical and hippocampal inputs at LA underlies fear renewal likely through inactivation of a GABA_B_R-mediated heterosynaptic inhibition that acts as a gatekeeper for the cross-talk of the convergent inputs. Consistent with this idea, damage to presynaptic GABA_B_ signaling has been implicated in generalization of conditioned fear [59].

In summary, we identified synaptic adaptations of the ACx → LA pathway through the vHPC → LA projections that function as a master switch to suppress or express the learned fear, unveiling a previously unknown circuit that specifically governs the fate of fear memory. The demonstrated synaptic associativity between the two pathways represents a major conceptual framework for understanding synaptic mechanisms of context-specific relapse of conditioned fear after extinction. Mechanistic elucidation of such presynaptic associativity for LTP would further inspire novel promising strategies for managing excessive traumatic memory and adaptive behaviors.

## MATERIALS AND METHODS

For details, see supplementary data.

## Supplementary Material

nwab004_Supplemental_File
