# Supplementary material for: Input associativity underlies fear memory renewal
Source: Natl Sci Rev. 2021 Jan 8;8(9):nwab004. doi: 10.1093/nsr/nwab004 (PMC8433092; doi:10.1093/nsr/nwab004)
Supplement: nwab004_Supplemental_File [file nwab004_supplemental_file.pdf]

Supplementary Data for

Input associativity underlies fear memory renewal

Wei-Guang Li[1,2,†,*], Yan-Jiao Wu[1,2,†], Xue Gu[1,2,†], Hui-Ran Fan[2], Qi Wang[1,2], Jia-Jie Zhu[2], Xin Yi[1,2], Qin Wang[2], Qin Jiang[1,2], Ying Li[1,2], Ti-Fei Yuan[3], Han Xu[4], Jiangteng Lu[1,2], Nan-Jie Xu[2], Michael Xi Zhu[5], Tian-Le Xu[1,2,6,*]

The file includes:

Material and methods

Supplementary Figs. 1 to 11

MATERIALS AND METHODS

Mice

All of the animal procedures were approved by the Animal Ethics Committee of Shanghai Jiao Tong University School of Medicine and by the Institutional Animal Care and Use Committee (Department of Laboratory Animal Science, Shanghai Jiao Tong University School of Medicine; Policy Number DLAS-MP-ANIM. 01–05). All behavioral measurements were performed in awake, unrestrained, mice (male, 8–12 weeks old, C57BL/6J background). The C57BL/6J mice were purchased from Shanghai Laboratory Animal Center at the Chinese Academy of Sciences (Shanghai, China). The $Fos^{2A\text{-}iCreER}$ (TRAP2) (stock no. 030323) mice were purchased from the Jackson Laboratory (Maine, USA). The lox-stop-lox-H2B-GFP ($H2B\text{-}GFP^{flox}$) and intersection-subtraction (IS) reporter mice were the generous gifts of Prof. Miao He (Fudan University, Shanghai, China). All behavioral measurements were performed by raters blinded to the experimental groups. All mice were group-housed on a 12-h light/dark cycle with rodent chow and water *ad libitum*. All experimental manipulations were performed during the light-on phase of the cycle in accordance with the institutional guidelines.

Fear conditioning, extinction, and renewal

All auditory fear conditioning, extinction, and renewal procedures were performed using the Ugo Basile Fear Conditioning System (UGO BASILE srl) with modification of a previous study (1). Briefly, mice were first handled and habituated to the

conditioning chamber for five consecutive days. The conditioning chambers (17 cm × 17 cm × 25 cm) equipped with stainless-steel shocking grids were connected to a precision-feedback current-regulated shocker (UGO BASILE srl). During fear conditioning, the chamber walls were covered with black-and-white checkered wallpaper, and the chambers were cleaned with 75% ethanol (context A). On day 0, mice were conditioned individually in context A with six pure tones (CS; 4 kHz, 76 dB, 20 s each) delivered at variable intervals (20–180 s), and each tone was co-terminated with a foot shock (US; 0.5 mA, 2 s each). ANY-maze software (Stoelting Co.) was used to automatically control the delivery of tones and foot shocks. Conditioned mice were returned to their home cages 30 s after the end of the last tone, and the floor and walls of the cage were cleaned with 75% ethanol for each mouse. For extinction learning, on days 1 and 2, mice trained in context A with six CS-US pairings on day 0 were presented with 12 CS presentations (4 kHz, 76 dB, 30 s each) without foot shock in a test chamber which had gray non-shocking plexiglass floor and dark gray wallpapers and was cleaned with 4% acetic acid solution (context B). On day 3, mice received four CS-alone (30 s each) presentations in the extinction context (context B) for extinction test (also referred to as the ABB test) or in the conditioning context (context A) for fear renewal (also referred to as ABA renewal). For the fear generalization test, mice were presented with the unconditioned white noise (76 dB, 30 s each) in the context as indicated in the Figures. For additional extinction training in the renewal context, mice experienced with fear renewal were then presented with 12 CS presentations (4 kHz, 76 dB, 30 s each) without foot shock in the context A (also referred to as the ABA

extinguished), while the control group was kept in the homecage without any treatment (also referred to as the ABA homecage). During behavioral testing, the chamber was placed in a sound-attenuating enclosure with a ventilation fan and a single house light (UGO BASILE srl). The movement of the mouse in the conditioning or test chamber was recorded using a near-infrared camera and analyzed in real-time with ANY-maze software. The ANY-maze behavior tracking software uses freezing score to represent the freezing status of the animal. The freezing score is a unit-less value as a result of rather complex calculations. When the software is calculating the freezing score, it looks for animal movements in the entire apparatus, which is accomplished by comparing every pixel of the current frame to earlier ones. If the software fails to find any movement (large number of flickering pixels) in the apparatus, the animal would be considered to be freezing. The software also includes 'noises' of the video when calculating the freezing score, for example, a breathing animal would cause some pixels to flicker. These kinds of noises coming with animals' physiological activities would have an influence on the value of the freezing score. Typically, louder video noise would result in lower freezing score of the animal at that frame. Finally, the software would give a result of freezing score at each frame, and periods that the animal is considered to be freezing according to the threshold setting. Freezing was defined as the absence of all movement, except respiration, for at least 2 s and was scored automatically using ANY-maze software. For animals connected to an optical fiber to the head, light stimuli during test sessions can interfere with the program's motion detection, so freezing of these sessions was scored independently for each video by an experienced experimenter

in a double-blind manner. The time spent freezing during the tone (cue) was measured for each tone presentation.

Virus constructs

The following viruses were used: AAV-retro-Syn-mCherry (Serotype 2/retro), AAV-retro-Syn-Cre-mCherry (Serotype 2/retro), AAV-CaMKIIα-eNpHR3.0-EYFP (Serotype 2/9), AAV-CaMKIIα-EYFP (Serotype 2/9), AAV-CaMKIIα-ChR2-E123T/T159C-mCherry (Serotype 2/9), AAV-CaMKIIα-mCherry (Serotype 2/9), AAV-CaMKIIα-ArchT-GFP (Serotype 2/9), AAV-CaMKIIα-GFP (Serotype 2/9) were purchased from Obio Technology Co. Ltd. (Shanghai); AAV-EF1α-DIO-TetTox-GFP and AAV-EF1α-DIO-GFP (Serotype 2/9) were packaged by Shanghai SunBio Biomedical technology Co. Ltd. (Shanghai); AAV-CaMKIIα-Chrimson-tdTomato, AAV-DIO-Chrimson-tdTomato, and AAV-CaMKIIα-Chronos-GFP (Serotype 2/9) were produced by Shanghai Taitool Bioscience Co. Ltd. (Shanghai); AAV-Syn-Cre (Serotype 2/1) and AAV-EF1α-Flp (Serotype 2/1) were produced by BrainVTA (Wuhan). All viral vectors were stored in aliquots at –80 °C until use. The viral titers for injection were more than 10^{12} viral particles per ml.

Viral injection

Mice at 6–7 weeks old were anesthetized with 1% sodium pentobarbital *via* a single intraperitoneal injection per mouse (10 ml per kg body weight), after which each mouse was mounted in a stereotactic frame with non-rupture ear bars (RWD Life Science).

After making an incision to the midline of the scalp, small bilateral craniotomies were performed using a microdrill with 0.5-mm burrs. Glass pipettes, with tip diameter of 10–20 μm were made with a P-97 Micropipette Puller (Sutter Glass pipettes) for AAV microinjections. The microinjection pipettes were first filled with silicone oil and then connected to a microinjector pump (KDS 310, KD Scientific) with full air exclusion. AAV-containing solutions were loaded into the tips of pipettes and injected at the following coordinates (2) (posterior to Bregma, AP; lateral to the midline, ML; below the Bregma, DV; in mm): LA: AP, –1.82 mm; ML, ±3.20 mm; DV, –4.20 mm; ACx: AP, –2.5 mm; ML, ±4.0 mm; DV, –2.3 mm; SCx: AP, –1.0 mm; ML, ±3.0 mm; DV, –1.5 mm; vHPC: AP, –3.16 mm; ML, ±2.90 mm; DV, –4.0 mm. Virus-containing solutions were injected bilaterally into the LA (0.3 μl/side), ACx (0.5 μl/side), SCx (0.5 μl/side), or vHPC (0.5 μl/side) at a rate of 0.1 μl/min. After injection, the pipette was left in place for an additional 10 min to allow the injectant to diffuse adequately.

In order to characterize the LA projectors in different brain regions (Fig. 2A–F), AAV-retro-Syn-mCherry was unilaterally injected into the LA. To silence vHPC LA projectors, AAV-retro-Syn-Cre-mCherry was unilaterally injected into the LA while AAV-EF1α-DIO-TetTox-GFP or AAV-EF1α-DIO-GFP (as the control) were bilaterally injected into the vHPC (Fig. 4A–C). To corroborate the hippocampal and cortical inputs into the amygdala and examine the convergence of these inputs, AAV1-Syn-Cre and/or AAV1-EF1α-Flp were unilaterally injected into ACx and/or the vHPC of IS mice (3, 4) (Fig. 2G–L and Supplementary Fig. 6). To allow optical stimulation of excitatory

neurons in the vHPC (Fig. 3A–H and Supplementary Figs. 7A–C), ACx (Fig. 3I–P), SCx (Supplementary Fig. 8A–D), or LA (Supplementary Fig. 1I–N), AAV-CaMKIIα-ChR2-E123T/T159C-mCherry was bilaterally injected into these regions. To enable optical excitation of excitatory neurons in SCx and ACx (Supplementary Fig. 8E–M) or in vHPC and ACx (Supplementary Fig. 10) independently (5), AAV-CaMKIIα-Chrimson-tdTomato and AAV-CaMKIIα-Chronos-GFP were injected into these areas, respectively, in a counterbalanced manner. To investigate the synaptic connections of ACx → LA projections under the conditions of whether or not vHPC → LA projections were silenced (Fig. 5), AAV-retro-Syn-Cre-mCherry was bilaterally injected into the LA, a mixture of AAV-EF1α-DIO-TetTox-GFP or AAV-EF1α-DIO-GFP (as the control) plus AAV-DIO-Chrimson-tdTomato was bilaterally injected into the vHPC, while AAV-CaMKIIα-Chronos-GFP was bilaterally injected into ACx.

To enable optical excitation of excitatory inputs of ACx and vHPC to LA independently (Fig. 6A and B) (5), AAV-CaMKIIα-Chrimson-tdTomato and AAV-CaMKIIα-Chronos-GFP were injected into these areas. To avoid the influence of different channelrhodopsins and/or light wavelengths on the efficiency of light-induced excitation, a half of the animals received AAV-CaMKIIα-Chrimson-tdTomato in ACx and AAV-CaMKIIα-Chronos-GFP in vHPC as illustrated in Fig. 6, while another half received AAV-CaMKIIα-Chronos-GFP in ACx and AAV-CaMKIIα-Chrimson-tdTomato in vHPC, The results were combined in the summary data (Fig. 6B lower panels). Mice were allowed to recover for at least 4–6 weeks before behavioral and

other tests. The injection sites were examined after experiments by the expression of the fluorescent proteins, GFP, YFP, mCherry, or tdTomato.

Neuronal tagging

Recombination was induced with 4-hydroxytamoxifen (4-OHT, Sigma-Aldrich, Catalog no. H6278) according to previous studies (6-9) with modifications. In brief, the 4-OHT was dissolved at 20 mg/ml in ethanol by shaking at 37 °C for 15 min and was then aliquoted and stored at –20 °C for up to several weeks. Before use, 4-OHT was redissolved in ethanol by shaking at 37 °C for 15 min, corn oil (Sigma-Aldrich, Catalog no. C8267) was added to give a final concentration of 10 mg/ml 4-OHT, and the ethanol was evaporated by vacuum under centrifugation. The final 10 mg/ml 4-OHT solutions were stored for at most 24 h at 4 °C before use. All injections were delivered intraperitoneally (i.p.). Mice were transported from the vivarium to an adjacent holding room at least 3 h before the 4-OHT injections to minimize transportation-induced immediate early gene activity. Activity-dependent neuronal tagging was induced by a single intraperitoneal injection of 4-OHT (20 mg/kg mice) administered immediately after fear conditioning for the fear acquisition-tagged mice. Mice were then returned to the vivarium with a regular 12 h light-dark cycle for the remainder of the experiment.

Optogenetic manipulations during extinction test or fear renewal

To investigate optogenetic-mediated effects during either extinction test or fear renewal, mice were subjected to auditory fear conditioning and to two consecutive days of

extinction training. To test for effects of light-induced activation of LA neurons during extinction testing (Supplementary Fig. 1I-N), mice were implanted with light-emitting diode (LED) optical connectors (200 μm O.D., N.A. = 0.37, λ = 473 nm) into the LA at two weeks after viral injections. An external receiving end was used to connect a 473-nm wireless optogenetic system (Hangzhou Newdoon Technology Co. Ltd) to the implanted LED optical connectors in each mouse. The light pulses were controlled in coordination with a fear conditioning system (UGO BASILE srl), where blue light (473 nm, 4–6 mW) was delivered in 10-ms pulses at 20 Hz during presentation of every 30-s CS (exceeding 5 s before and after the CS to ensure the light delivery covered the CS exposure for the extinction test).

To test for effects of light-induced inhibition of LA neurons during fear renewal (Supplementary Fig. 1E–H), different types of LED optical connectors (200 μm O.D., N.A. = 0.37, λ = 532 nm) and external receiving ends to connect the 532-nm wireless optogenetic system (Hangzhou Newdoon Technology Co. Ltd) were used to deliver continuous green light (532 nm, 4–6 mW) during presentation of every 30-s CS (exceeding 5 s before and after the CS to ensure the light delivery covered the CS exposure for the fear renewal test). For optogenetic inhibition of ACx → LA projections (Fig. 4D–F), the wired optogenetic system was used. An external optical fiber was used to connect a 589-nm laser power source (Changchun New Industries Optoelectronics Technology Co., Ltd., China) to the implanted optical fiber (200 mm O.D., N.A. = 0.37) in each mouse. The external optical fiber was attached to a rotary joint (FRJ_1 × 1_FC-

FC, Doric Lenses) to allow the mouse to freely behave. The test mouse was allowed to habituate in its home cage with the external fiber attached for at least 10 min. Laser pulses were controlled through a customized MATLAB program (AniLab Software and Instruments). For optogenetic inhibition of ACx terminals in the LA, yellow light (589 nm) was delivered in a continuous pattern during presentation of every 30-s CS (exceeding 5 s before and after the CS to ensure the light delivery covered the CS exposure for the fear renewal test), with the final output power ranging from 8–10 mW depending on the light transmission efficacy of the optical fiber used.

To allow light-induced activation of vHPC → LA or ACx → LA projections during memory test in either extinction context or conditioning context in the same animal (Fig. 6A and B), we designed an optogenetic system that allows switching of light sources (473-nm and 638-nm LED, Hangzhou Newdoon Technology Co. Ltd) for connection to the same optical fiber (200 mm O.D., N.A. = 0.37) implanted into the LA area of the mouse that received desired AAV carrying different channelrhodopsins in ACx and vHPC. To photostimulate either vHPC → LA or ACx → LA inputs in the extinction context, the light pulses were coordinated with a fear conditioning system (UGO BASILE srl), where either blue (473 nm, 10-12 mW) or red light (638 nm, 15-20 mW) was delivered in 10-ms pulses at 20 Hz during the presentation of each 30-s CS (with 5 s added before and after the CS to ensure that the light delivery covers the CS exposure). To photostimulate either vHPC → LA or ACx → LA inputs in the renewal context (same as conditioning), the light pulses were coordinated the same way as

described above but the CS was omitted.

Cannula implantations and local drug injections

Mice were anaesthetized with 1% sodium pentobarbital and were then each fixed on a stereotaxic apparatus (RWD Life Science). Stainless-steel guide cannulae (RWD Life Science) were bilaterally implanted into the target brain areas, and the tips of cannulae were targeted at the following coordinates (in mm): LA: AP, –1.82 mm; ML, ±3.20 mm; DV, –3.20 mm. The cannulae were fixed to the skull using acrylic cement and two skull screws. Stainless-steel obturators (33 gauges) were inserted into guide cannulae to avoid obstruction until drug infusion. Mice were allowed to recover from surgery for two weeks before behavioral tests. Mice were handled and habituated to the infusion procedure several days before drug injections. During drug infusions, mice were briefly head-restrained, while the stainless-steel obturators were removed and injection cannulae (33 gauges, RWD Life Science) were inserted into the guide cannulae. Injection cannulae protruded 1.00 mm from the tips of guide cannulae. Infusion cannulae were connected *via* PE20 tubing to a microsyringe driven by a microinfusion pump (KDS 310, KD Scientific). Drugs were infused bilaterally into the target brain areas at a flow rate of 0.15 μl per min. After finishing drug injections, the injection cannulae were left in place for 2 min to allow the solution to diffuse from each cannula tip. The stainless-steel obturators were subsequently reinserted into guide cannulae and the mice returned to their home cage for 30 min before behavioral tests. A mixture (0.5 μl per side) of CNQX (10 mM in aCSF) and D-APV (12.5 mM in aCSF), picrotoxin

(100 μM in aCSF, 0.5 μl per side), or their respective vehicles were bilaterally microinfused into the LA (Supplementary Fig. 3). The injection sites were examined at the end of the experiments, and mice with incorrect diffusion scopes were excluded from further data analysis.

Slice electrophysiology

Whole-cell recordings were performed in acute brain slices from behaviorally trained mice and/or those that had been stereotaxically injected with AAV-CaMKIIα-ArchT-GFP, AAV-CaMKIIα-ChR2-mCherry, or AAV-CaMKIIα-eNpHR-EYFP in different brain regions according to a previous study (1). Mice were deeply anesthetized with 1% sodium pentobarbital and were subsequently decapitated. Brains were dissected quickly and were chilled in well-oxygenated (95% O_2/5% CO_2, v/v) ice-cold artificial cerebrospinal fluid (aCSF) containing the following (in mM): 125 NaCl, 2.5 KCl, 12.5 D-glucose, 1 $MgCl_2$, 2 $CaCl_2$, 1.25 NaH_2PO_4, and 25 $NaHCO_3$ (pH 7.35-7.45). Coronal brain slices (300-μm thick) containing regions of interest were cut with a vibratome (Leica VT1000S, Germany). After recovery for 1 h in oxygenated aCSF at 30 ± 1°C, each slice was transferred to a recording chamber and was continuously superfused with oxygenated aCSF at the rate of 1–2 ml per minute. The principal neurons in ACx or those in LA were patched under visual guidance using infrared differential-interference contrast microscopy (BX51WI, Olympus) and an optiMOS camera (QImaging). The slices were continuously perfused with well-oxygenated aCSF at 35 ± 1°C during all electrophysiological studies. Whole-cell patch clamp recordings were performed using

an Axon 200B amplifier (Molecular Devices). Membranous currents were sampled and analyzed using a Digidata 1440 interface and a personal computer running Clampex and Clampfit software (Version 10, Axon Instruments). Access resistance was 15–30 MΩ and only cells with a change in access resistance < 20% were included in the analysis. Optical stimulation of ChR2-/ Chronos-, ArchT-, eNpHR-, or Chrimson-expressing neurons was performed using a collimated LED (Lumen Dynamics) with peak wavelengths of 473, 532, 589, 638 nm, respectively. The LED was connected to an Axon 200B amplifier to trigger photostimulation. The brain slice in the recording chamber was illuminated through a 40 × water-immersion objective lens (LUMPLFLN 40XW, Olympus). The intensity of photostimulation was directly controlled by the stimulator (2–18 mW/mm^2), while the duration was set through Digidata 1440 and pClamp 10.5 software. The functional potency of the ChR2-expressing virus was validated by measuring the number of action potentials elicited in LA neurons using different frequencies of blue-light stimulation (1 ms, 5, 10 and 20 Hz) and the inward photocurrents (1 s pulse) mediated by ChR2 in brain slices (Supplementary Fig. 1J–M). To corroborate the functional potency of ArchT- (Supplementary Fig. 1G) or eNpHR-mediated (Supplementary Fig. 9A–C) optogenetic inhibition, green (λ = 532 nm) or yellow light (λ = 589 nm) was delivered to generate outward photocurrents (1-s pulse) under voltage-clamp mode and to promote membrane hyperpolarization and to reduce spikes to current injection under current-clamp mode.

Spontaneous excitatory postsynaptic currents. For electrophysiological recordings

of synaptic adaptions associated with fear renewal in LA slices, immediately after either extinction testing (ABB test) or fear renewal (ABA renewal), the mice were sacrificed for slice preparation. For recordings of spontaneous excitatory postsynaptic currents (sEPSCs, Supplementary Figs. 2H–J, 11C–E) in LA neurons, the holding potential was –70 mV. Patch pipettes had open-tip resistances of 3–5 MΩ when filled with an intracellular solution that contained the following (in mM): 132.5 cesium gluconate, 17.5 CsCl, 2 $MgCl_2$, 0.5 EGTA, 10 HEPES, 4 Mg-ATP, and 5 QX-314 chloride (280–300 mOsm, pH 7.2 adjusted with CsOH). The baseline sEPSCs of LA neurons were recorded for 5 min and were analyzed from 100–200 s after the establishment and stabilization of the recording. Data were analyzed using the Mini-analysis Program (Synaptosoft) with an amplitude threshold of 5 pA.

Light-evoked EPSCs. To evoke synaptic responses in the LA by optogenetic photostimulation of vHPC, ACx, or SCx axons, each slice was illuminated every 20 s with blue- or red-light pulses of 5-ms durations according to the light sensitivity of the expressed opsin type (ChR2, blue, $\lambda = 473$ nm; Chrimson, red, $\lambda = 638$ nm; Chronos, blue, $\lambda = 473$ nm). In order to prevent polysynaptic activities from being detected in EPSC recordings, we applied appropriate photostimulation intensities that produced 30–50% of the maximal synaptic response. For recording light-evoked EPSCs, the recording pipettes (3–5 MΩ) were filled with a solution containing the following (in mM): 132.5 cesium gluconate, 17.5 CsCl, 2 $MgCl_2$, 0.5 EGTA, 10 HEPES, 4 Mg-ATP, and 5 QX-314 chloride (280–300 mOsm, pH 7.2 with CsOH). To determine the paired-

pulse ratio (PPR), the patched LA neurons were voltage clamped at –70 mV. The AMPAR oEPSCs were evoked by paired photostimulations (25, 50, 100, 200, and 500 ms intervals; 5-ms duration) of opsin-expressing axons and PPRs were calculated as the peak amplitude ratio of the second to the first oEPSC (Figs. 3C, D, K, L, 5G–J, and Supplementary Figs. 8, 11F–I). To determine the NMDAR/AMPAR ratio (Fig. 3E, F, M, and N), the AMPAR-mediated oEPSCs were recorded at –70 mV while the NMDAR-mediated oEPSCs were recorded in the presence of CNQX (20 μM) and picrotoxin (100 μM) at +40 mV. For each LA neuron, photostimulations of the same intensity and duration were used to record the AMPAR- and NMDAR-mediated oEPSCs. Likewise, the NMDAR-mediated oEPSCs at different voltages (by incremental increases of voltage, –80 to +40 mV, in 20 mV increments; Fig. 3G, H, O, and P) were also recorded in the presence of CNQX (20 μM) and picrotoxin (100 μM).

Electrical stimulation-evoked EPSCs. EPSCs were recorded from LA principal neurons with an Axon 200B amplifier (Molecular Devices), and the stimulations were delivered with a bipolar tungsten stimulating electrode (0.1-ms duration) placed within the ACx region (Supplementary Fig. 9D–G) to stimulate the auditory cortical glutamatergic inputs to the LA. The AMPAR-mediated EPSCs were induced by repetitive stimulations at 0.05 Hz, with the patched neuron was voltage-clamped at −70 mV. To determine the PPR, the patched LA neurons were voltage clamped at –70 mV.

Spike firing. Spiking activity (Supplementary Figs. 1B-D, G, L, M, 2B-G, 9C) and

related membrane properties of LA neurons were measured with an internal solution containing the following (in mM): 145 potassium gluconate, 5 NaCl, 10 HEPES, 2 MgATP, 0.1 Na_3GTP, 0.2 EGTA, and 1 $MgCl_2$ (280–300 mOsm, pH 7.2 with KOH). Data were analyzed by the Mini Analysis Program (Synaptosoft) with an amplitude threshold of 20 mV.

Histology and fluorescent immunostaining

Animals were deeply anesthetized with 1% sodium pentobarbital and were transcardially perfused with saline followed by ice-cold 4% paraformaldehyde (PFA) in phosphate-buffered saline (PBS). To examine LA projectors in different brain regions including the vHPC, dHPC, and ACx, AAV-retro-Syn-mCherry was injected into LA at four weeks prior to the perfusion. After dissection, coronal brain slices containing the whole vHPC, dHPC, ACx, and LA were sectioned (30-μm thicknesses) using a vibratome (VT1000S, Leica) and were processed for *post-hoc* analysis or verification of viral-infection efficiency and specificity. After a 15-min incubation in 4,6-Diamidino-2-phenylindole dihydrochloride hydrate (DAPI) solution (1: 1000), sections were washed three times (15 min each time) in PBS with 0.1% Tween-20. Slides were mounted in the dark with glass coverslips using mounting media. The coverslips were sealed to the slide with nail polish. Stained slides were prepared for microscopy. The mCherry and DAPI signals of the LA were observed using a fluorescent microscope. For quantification of mCherry labeling, we used ImageJ software (NIH Image, version 1.80e) to manually count fluorescent-positive cells. The numbers of mCherry-positive

cells were counted exhaustively from one out of every three slices covering the whole vHPC (Fig. 2A–C), ACx (Fig. 2D–F), and dHPC (Supplementary Fig. 5) regions based on their coordinates. All fluorescent images were collected by taking serial z-stack images through 10 × or 20 × objectives of a confocal microscope (Digital Eclipse A1R+, Nikon). Moreover, using standard histological methods and confocal microscopy, we also validated the locations of optical fiber tips for all optogenetic experiments, as well as those of cannula tips for all *in-situ* pharmacological behavioral experiments.

For the c-fos staining, brain slices were washed three times (5 min each time) with 1 × PBS and were then blocked with 10% normal goat serum in PBS for 1 h, after which they were incubated for 48 h at 4°C with rabbit anti-c-fos (1:1000, Cell Signaling Technology, catalog no. 2250). Sections were then washed with PBS, incubated in 2% normal goat serum for 10 min, and then incubated for 4 h with Alexa Fluor® 568 donkey anti-rabbit IgG (H+L) (ThermoFisher Scientific; catalog no. A10042). After staining, sections were rinsed in PBS and slides were mounted in the dark with glass coverslips using mounting media. The coverslips were sealed to the slides with nail polish. Stained slides were then observed via microscopy. All counts were performed blind with respect to treatment groups. In Fig. 1C–E, the amount of reactivation was normalized for chance overlap by dividing the percentage of colabeled$^+$ among DAPI$^+$ by chance (($GFP^+/DAPI^+$) × ($c\text{-}fos^+/DAPI^+$) × 100) (9-11).

QUANTIFICATION AND STATISTICAL ANALYSIS

Data are presented as the mean ± the standard error of the mean (S.E.M.) unless indicated otherwise. Most histograms display individual data points that represent the values and numbers of individual samples for each condition. Data distributions were tested for normality and variance equality among groups was assessed using the Levene's test. Statistical comparisons were performed using unpaired Student's *t* tests as well as one-way analyses of variance (ANOVAs) or two-way repeated-measures ANOVAs. For *post-hoc* analysis, we used Bonferroni's corrections for multiple comparisons. Statistical analysis was performed with IBM SPSS Statistics 25 (SPSS), and $p < 0.05$ was considered statistically significant. Significance is mainly displayed as $^{*}p < 0.05$, $^{**}p < 0.01$, $^{***}p < 0.001$, and in some cases is indicated as $^{\#}p < 0.05$, $^{\#\#}p < 0.01$, $^{\#\#\#}p < 0.001$ for multiple comparisons; N.S. denotes non-significant values.

DATA AVAILABILITY

All data needed to evaluate the conclusions in the paper are present in the paper and/or the Supplementary Data. Additional data available from authors upon request.

REFERENCES

1. Wang, Q, Wang, Q, Song, XL, *et al.* Fear extinction requires ASIC1a-dependent regulation of hippocampal-prefrontal correlates. *Sci Adv*. 2018; **4**(10): eaau3075.
2. Franklin, KBJ, Paxinos, G. *The mouse brain in stereotaxic coordinates. Third edition.* San Diego, CA: Academic Press; 2007.
3. He, M, Tucciarone, J, Lee, S, *et al.* Strategies and tools for combinatorial targeting

of GABAergic neurons in mouse cerebral cortex. *Neuron*. 2016; **91**(6): 1228-43.

4. Zingg, B, Chou, XL, Zhang, ZG*, et al.* AAV-mediated anterograde transsynaptic tagging: mapping corticocollicular input-defined neural pathways for defense behaviors. *Neuron*. 2017; **93**(1): 33-47.
5. Klapoetke, NC, Murata, Y, Kim, SS*, et al.* Independent optical excitation of distinct neural populations. *Nat Methods*. 2014; **11**(3): 338-46.
6. Guenthner, CJ, Miyamichi, K, Yang, HH*, et al.* Permanent genetic access to transiently active neurons via TRAP: targeted recombination in active populations. *Neuron*. 2013; **78**(5): 773-84.
7. Allen, WE, DeNardo, LA, Chen, MZ*, et al.* Thirst-associated preoptic neurons encode an aversive motivational drive. *Science*. 2017; **357**(6356): 1149-55.
8. DeNardo, LA, Liu, CD, Allen, WE*, et al.* Temporal evolution of cortical ensembles promoting remote memory retrieval. *Nat Neurosci*. 2019; **22**(3): 460-9.
9. Lacagnina, AF, Brockway, ET, Crovetti, CR*, et al.* Distinct hippocampal engrams control extinction and relapse of fear memory. *Nat Neurosci*. 2019; **22**(5): 753-61.
10. Reijmers, LG, Perkins, BL, Matsuo, N*, et al.* Localization of a stable neural correlate of associative memory. *Science*. 2007; **317**(5842): 1230-3.
11. Cai, DJ, Aharoni, D, Shuman, T*, et al.* A shared neural ensemble links distinct contextual memories encoded close in time. *Nature*. 2016; **534**(7605): 115-8.

Supplementary Figures and Legends

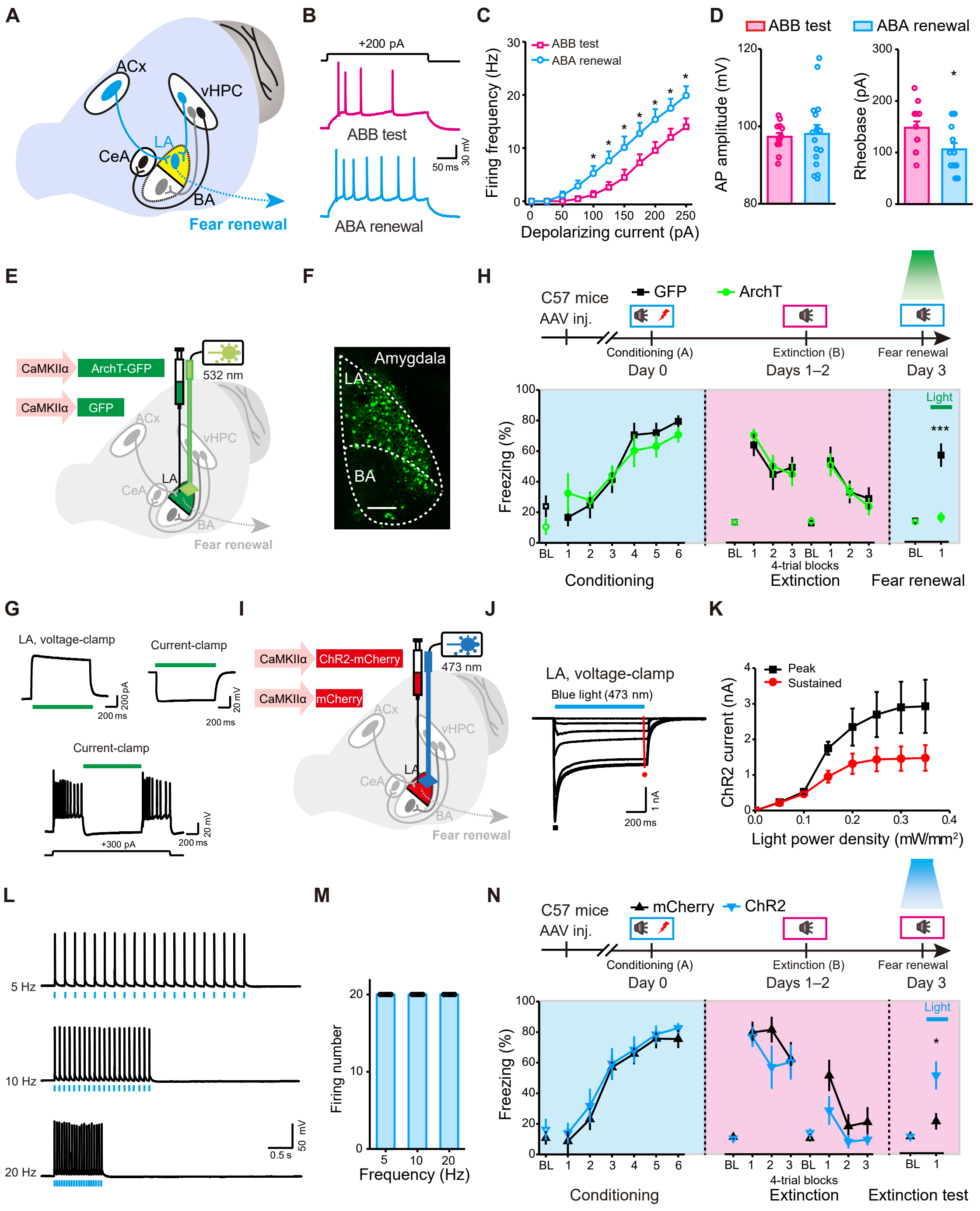

Supplementary Fig. 1. Fear renewal is associated with neuronal hyperactivity of the LA.

(A–D) Action potential (AP) firing of principal neurons in the LA to depolarizing current injections recorded *ex vivo*. (A) Schematic of a potential engagement of the lateral amygdala (LA) in fear renewal. ACx, auditory cortex; vHPC, ventral hippocampus; CeA, central amygdala; BA, basal amygdala. (B) Representative traces

showing voltage responses of principal neurons in LA to 500-ms-step injections of current of +200 pA. (C) The frequency of AP discharge as a function of step-current intensity (0–250 pA, 500 ms). Statistics are as follows: two-way repeated measures ANOVA, main effect of behavior, $F_{1,288} = 39.742$, $p < 0.001$; $^{*}p < 0.05$ between samples of the same intensity, unpaired Student's *t*-test. ABB test, n = 13 neurons of seven mice; ABA renewal, n = 16 neurons of seven mice. (D) Summary data for AP amplitude and rheobase obtained from the slice recording data set shown in (C).

(E–H) Effects of optogenetic silencing of LA principal neurons on fear renewal. (E) Experimental schemes. (F) Representative image of GFP expression (*green*) in a mouse that received AAV-CaMKIIα-ArchT-GFP injection into the LA. Scale bar, 200 μm. (G) Representative traces of green-light-evoked (1 s, continuous) outward current (upper, *left*), membrane hyperpolarization (upper, *right*), and blockade of AP firing (lower) recorded from ArchT-GFP-expressing neurons. (H) Upper, behavioral protocols. Lower, time course of freezing responses to the context only (baseline, BL) or CS. While the freezing response during conditioning was calculated by percent freezing time to CS in individual trials, those during extinction learning and fear renewal were calculated by the average freezing responses of four consecutive trials. The same convention was used for the calculation of all behavioral results. Statistics are as follows: two-way repeated measures ANOVA, main effect of AAV, conditioning, $F_{1,106} = 0.051$, $p = 0.821$; extinction learning, $F_{1,106} = 0.000$, $p = 0.990$; $^{***}p < 0.001$, unpaired Student's *t*-test. GFP, n = 10, ArchT, n = 8.

(I–N) Effects of optogenetic activation of LA principal neurons on extinction test. (I) Experimental schemes. (J–M) Electrophysiological verification of ChR2 expression in LA principal neurons. (J) ChR2-mediated currents evoked by 1-s pulses of light (473 nm, blue horizontal bar) with variable intensities at –70 mV in the voltage-clamp mode. (K) Input-output curves of ChR2-mediated photocurrents showing peak (*black square*) and sustained current components (*red circles*) plotted as a function of light intensity (light power density, mW/mm^2, n = 5 neurons from two mice). (L) APs evoked in a principal neuron in the LA in current-clamp mode by 1-ms pulses of photostimuli at different frequencies (blue vertical lines). (M) Summary plot of AP firing probability versus photostimulation frequency (n = 5 neurons of two mice). (N) Upper, behavioral protocols. Lower, time course of freezing responses to the context only (baseline, BL) or CS. Statistics are as follows: two-way repeated measures ANOVA, main effect of AAV, conditioning, $F_{1,94} = 1.184$, $p = 0.280$; extinction learning, $F_{1,94} = 5.171$, $p = 0.026$; $^{*}p < 0.05$, unpaired Student's *t*-test. mCherry, n = 9, ChR2, n = 7.

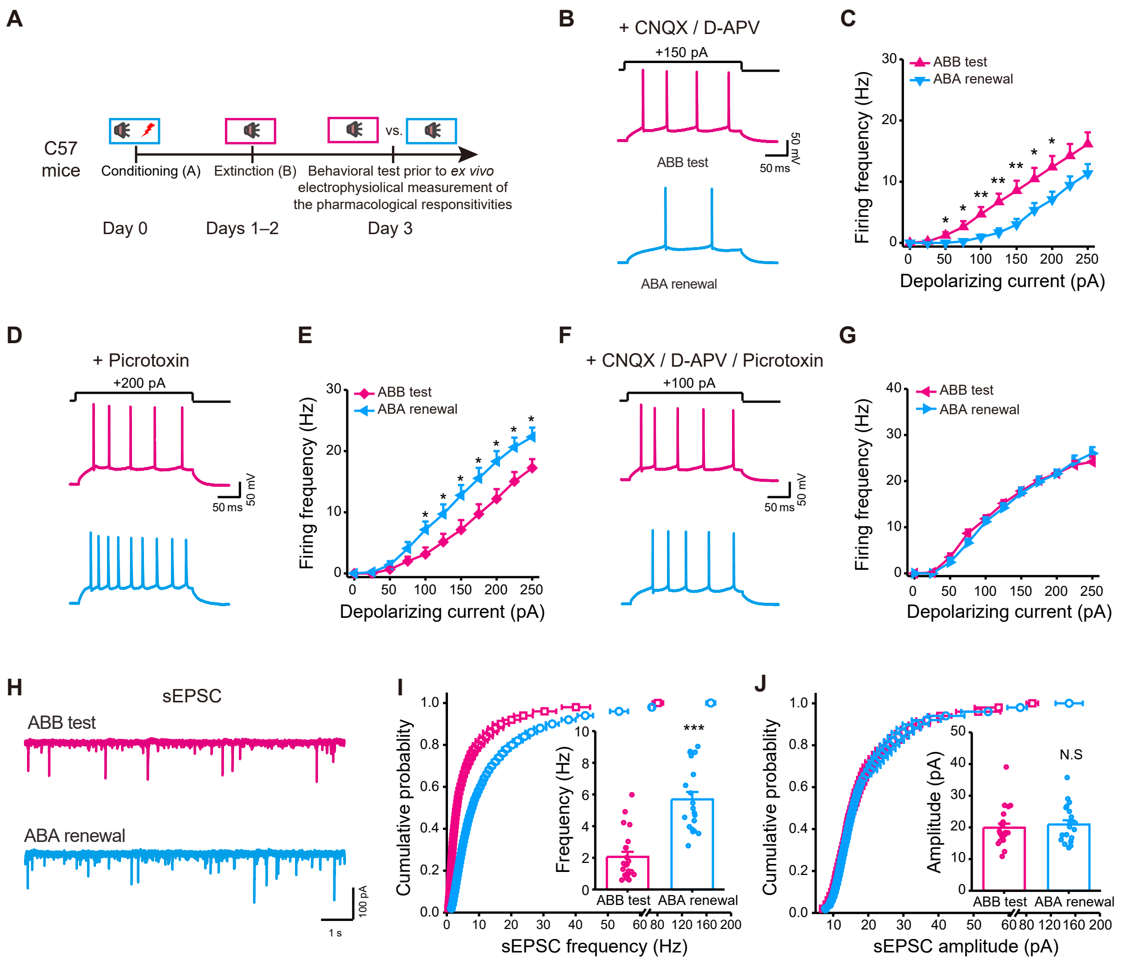

Supplementary Fig. 2. Roles of synaptic inputs on the LA excitability associated with fear renewal.

(A–G) Effects of synaptic antagonism on AP firing of principal neurons in the LA. Slice recordings were performed in a similar manner as in Supplementary Fig. 1B–D, but in the presence of AMPAR and NMDAR antagonists, CNQX (20 μM) and D-APV (50 μM), respectively (B and C), the presence of GABA$_A$R antagonist, picrotoxin (100 μM) (D and E), or CNQX, D-APV, and picrotoxin together (F and G). (A) Experimental schemes. (B, D, and F) Representative traces showing voltage responses of principal neurons in the LA to step injections of current as indicated. (C, E, and G) The frequency of AP discharge as a function of step-current intensity (0–250 pA, 500 ms). Statistics are as follows: two-way repeated measures ANOVA, main effect of behavior, (C) CNQX / D-APV, $F_{1,498} = 46.892$, $p < 0.001$; (E) picrotoxin, $F_{1,468} = 43.051$, $p < 0.001$; (G) CNQX / D-APV / picrotoxin, $F_{1,268} = 3.633$, $p = 0.058$. (C) ABB test + CNQX / D-APV, n = 26 neurons from seven mice; ABA renewal + CNQX / D-APV, n = 24 neurons from eight mice. (E) ABB test + picrotoxin, n = 21 neurons from six mice; ABA renewal + picrotoxin, n = 26 neurons from eight mice. (G) ABB test + CNQX / D-APV / picrotoxin, n = 12 neurons from three mice; ABA renewal + CNQX / D-APV / picrotoxin, n = 15 neurons from four mice.

(H–J) Fear-renewal-driven changes of sEPSCs (as global synaptic inputs) in LA

neurons. (H) Representative traces of sEPSCs in LA neurons. (I and J) Cumulative distribution plots for sEPSC frequency (I) and amplitude (J). The inset shows values for individual neurons and the summary data. N.S., no significant difference, $^{***}p < 0.001$, unpaired Student's *t*-test. ABB test, n = 22 neurons from eight mice; ABA renewal, n = 20 neurons from seven mice.

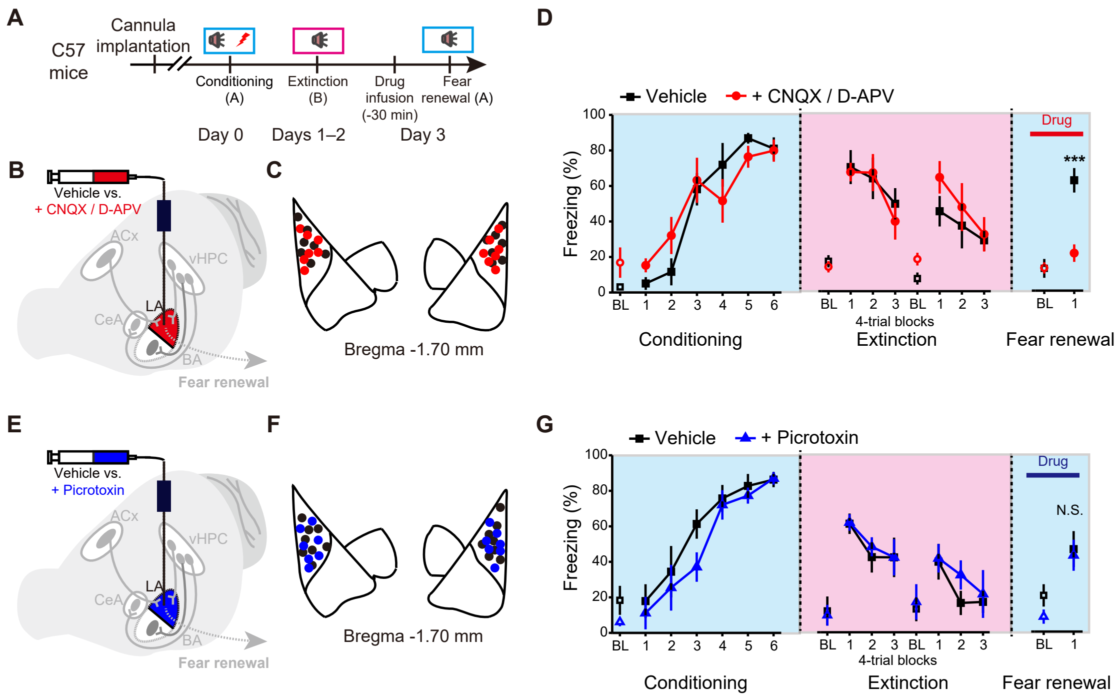

Supplementary Fig. 3. Effects of pharmacological inhibition of ionotropic glutamate or GABA receptors in LA on fear renewal.

(A) Experimental schemes.

(B and E) Schematics of cannula implantations and drug injections.

(C and F) Diagrams indicating sites of vehicle (aCSF, black) or CNQX (5 nmol) plus D-APV (6.25 nmol in aCSF, red) or picrotoxin (50 pmol, blue) infusion for all samples.

(D and G) Time courses of freezing responses to the context only (baseline, BL) or CS. Statistics are as follows: two-way repeated measures ANOVA, main effect of drug, (D) conditioning, $F_{1,82} = 0.017$, $p = 0.896$; extinction learning, $F_{1,82} = 0.467$, $p = 0.497$; (G) conditioning, $F_{1,88} = 2.513$, $p = 0.117$; extinction learning, $F_{1,88} = 0.928$, $p = 0.338$. N.S., no significant difference, $^{***}p < 0.001$, unpaired Student's *t*-test. (D) Vehicle, n = 7, CNQX / D-APV, n = 7; (G) Vehicle, n = 8, picrotoxin, n = 7.

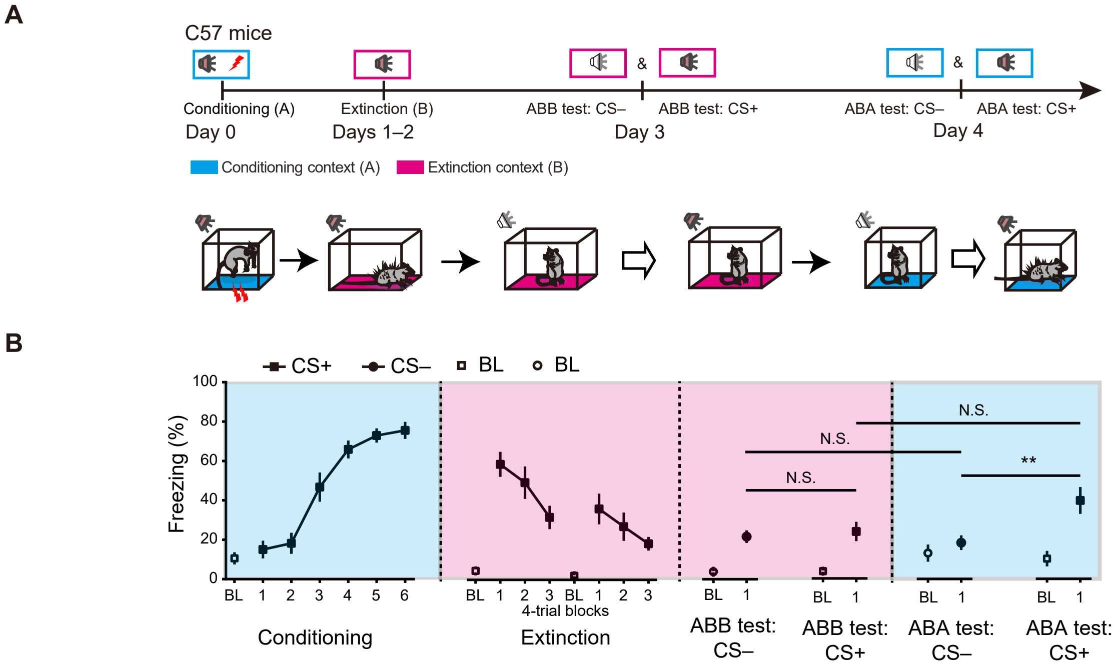

Supplementary Fig. 4. Fear renewal is selective for the specific fear memory acquired during conditioning.

(A and B) Presentation of an unconditioned tone failed to induce fear renewal. (A) Experimental schemes. Note the different tones, i.e. unconditioned tone (CS–) and conditioning tone (CS+), used during extinction test (Day 3) and fear renewal (Day 4). (B) Time course of freezing responses to the context only (baseline, BL) or CS– or CS+. N.S., no significant difference, $^{**}p < 0.001$, unpaired Student's *t*-test, compared as indicated. n = 10.

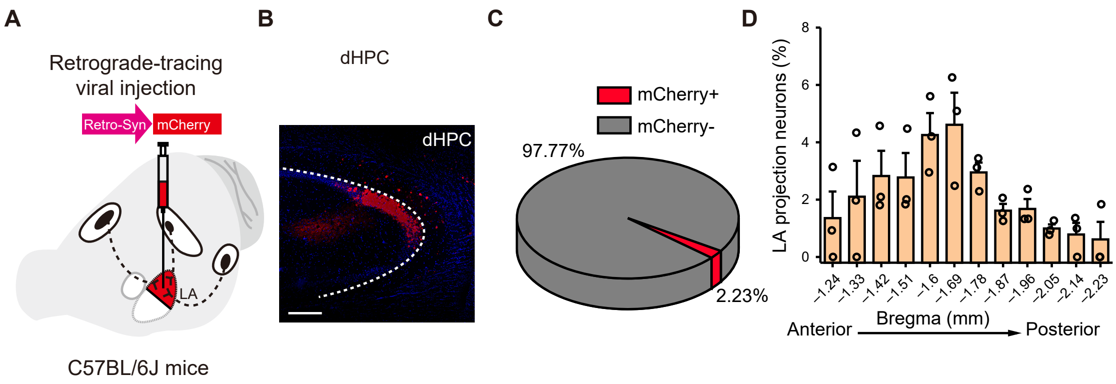

Supplementary Fig. 5. Quantification of LA-projecting neurons in dorsal hippocampus (dHPC).

(A) Schematics of AAV injection to identify LA-projecting neurons in upstream brain regions.

(B) Representative image of mCherry expression (*red*) in a mouse that received AAV-retro-Syn-mCherry injection into the LA as shown in Fig. 2B (*left*), showing a histological example of LA-projecting dHPC neurons (*red*, mCherry-positive). DAPI (*blue*) was used to label nuclei. Scale bar, 500 µm.

(C and D) Quantification of LA-projecting dHPC neurons. n = 3 mice.

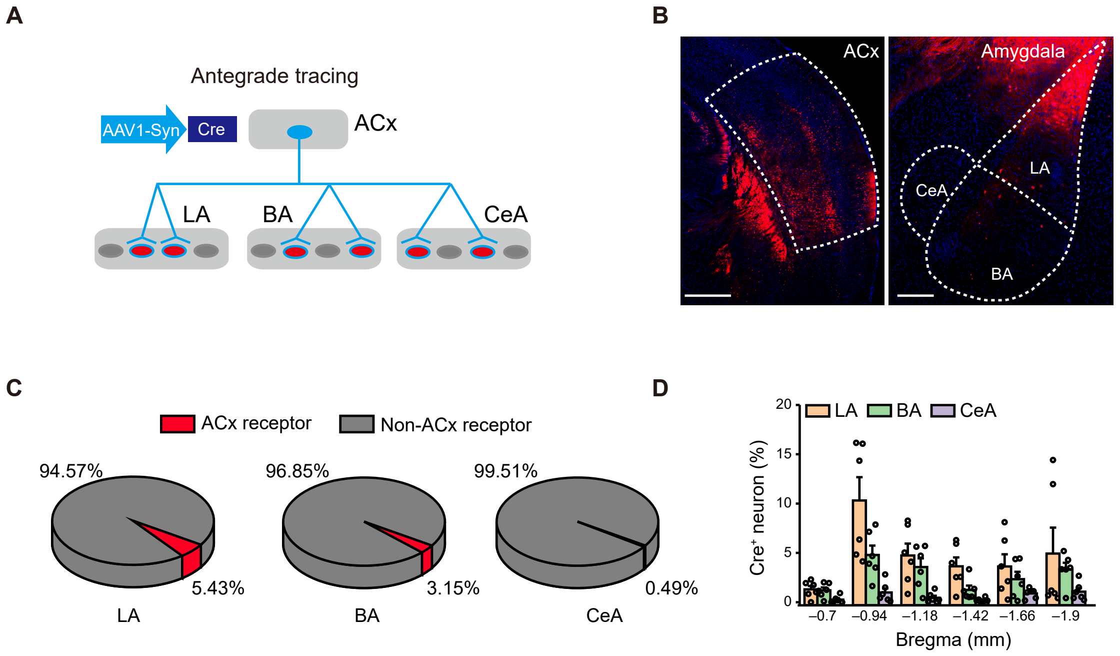

Supplementary Fig. 6. Anterograde transsynaptic mapping of amygdalar neurons receiving projections from ACx.

(A) Schematics of AAV injection to identify amygdalar neurons receiving projections from ACx.

(B) Representative images of tdTomato expression (*red*) in ACx (*left*) and the amygdala (*right*). DAPI (*blue*) was used to label nuclei. *Left*, ACx, scale bar, 500 μm; *Right*, amygdala, scale bar, 200 μm.

(C and D) Quantification of ACx receptors in LA, BA, and CeA. n = 6 mice.

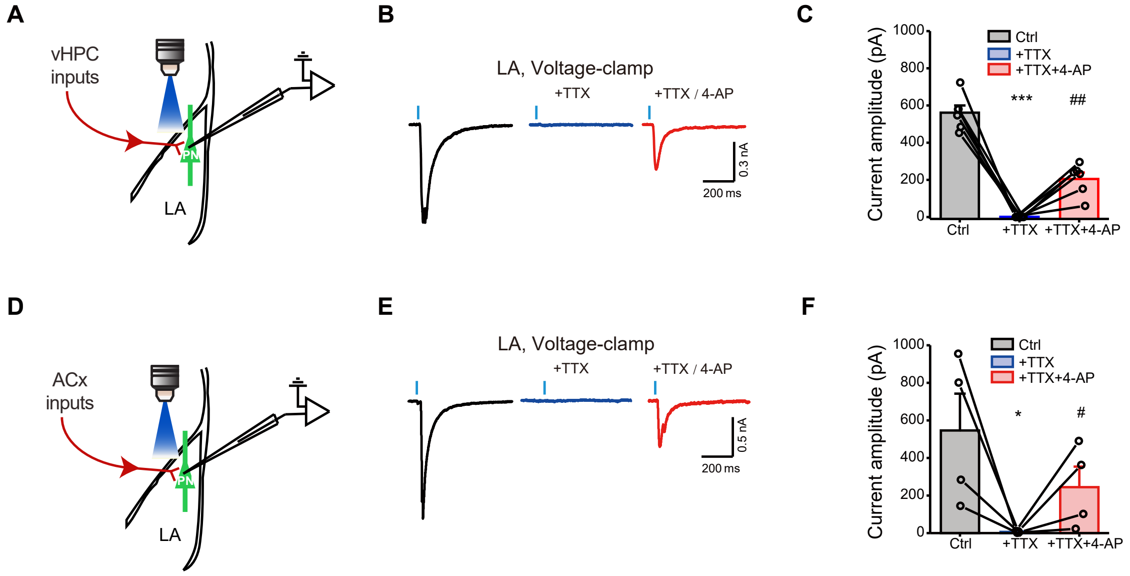

Supplementary Fig. 7. Electrophysiological characterization of vHPC → LA (A–C) and ACx → LA (D–F) projections.

(A and D) Experimental schemes.

(B and E) Representative traces of oEPSCs at vHPC → LA (B) or ACx → LA (E) synapses before and after bath application of TTX (1 μM), and TTX (1 μM) plus 4-AP (100 μM), respectively. The blue vertical bars above traces indicate the photostimulation (λ = 473 nm).

(C and F) Histograms of mean ± S.E.M. with circles denoting individual neurons. (C) n = 6 neurons from two mice. (F) n = 4 neurons from two mice. $^{*}p < 0.05$, $^{***}p < 0.001$, Ctrl *vs*. TTX; $^{\#}p < 0.05$, $^{\#\#}p < 0.01$, TTX *vs*. TTX plus 4-AP, paired Student's *t*-test.

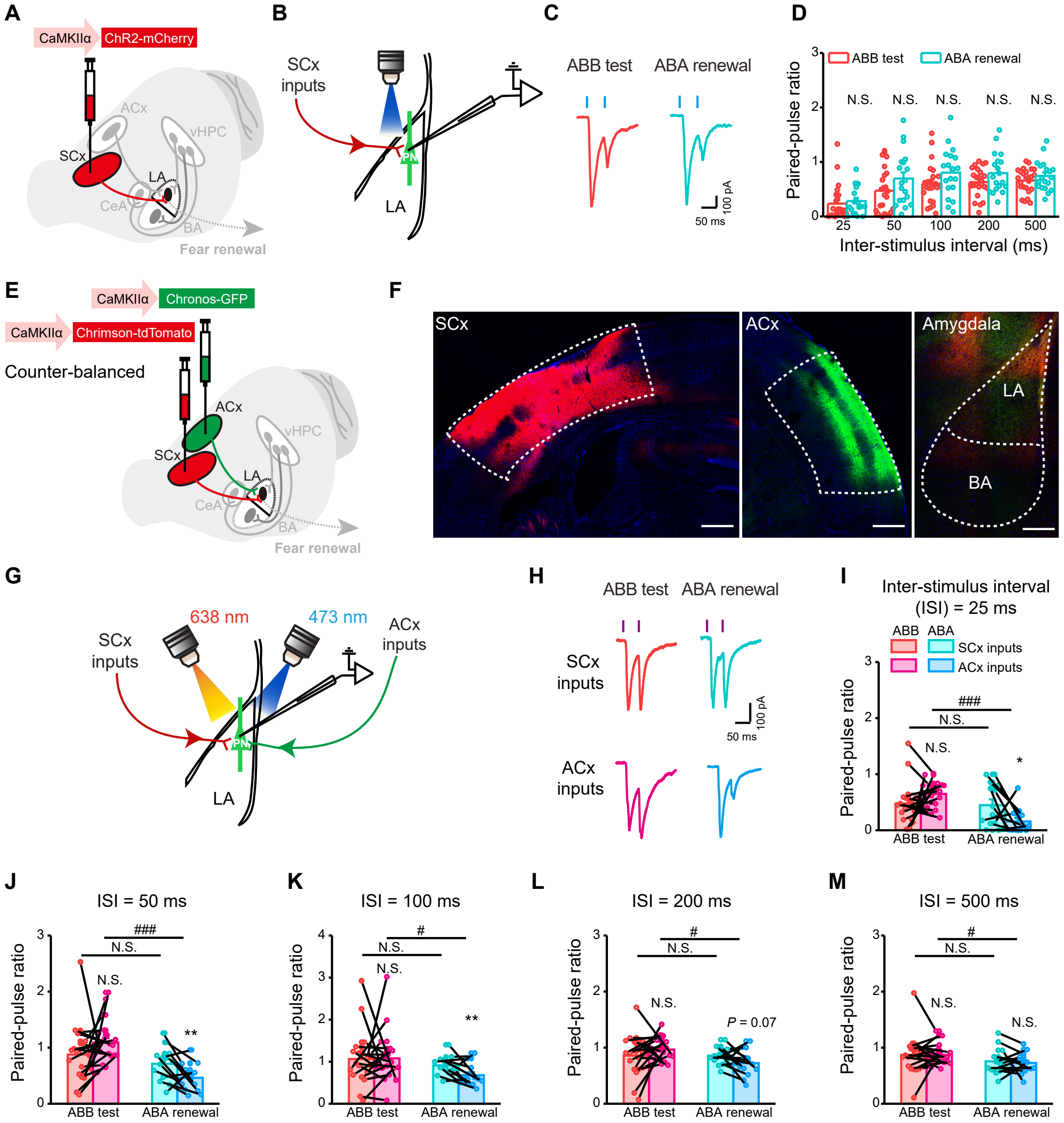

Supplementary Fig. 8. Effects of fear renewal on PPRs for oEPSCs at SCx → LA projections.

(A) Schematics of AAV injections.

(B) Experimental schemes.

(C) Representative traces of oEPSCs at SCx → LA synapses in extinction test and fear renewal groups induced by paired photostimulations (blue vertical bars) with 50-ms intervals.

(D) Histograms of mean ± S.E.M. with circles denoting PPRs of individual neurons. Statistics are as follows: two-way repeated measures ANOVA, main effect of behavior, $F_{1,213}$ = 12.464, p = 0.003; N.S., no significant difference, unpaired Student's *t*-test. ABB test, n = 24 neurons from eight mice; ABA renewal, n = 19 neurons from five mice.

(E–M) Comparison of PPRs for oEPSCs at SCx → LA and ACx → LA projections within the same neurons. (E) Schematics of AAV injections. (F) Representative images.

Left, SCx, scale bar, 500 μm; Middle, ACx, scale bar, 500 μm; *Right*, amygdala, scale bar, 200 μm. (G) Experimental schemes. (H) Representative traces of oEPSCs at SCx → LA and ACx → LA synapses recorded in the same neurons by paired photostimulations (violet vertical bars, 50-ms interval) of red- and blue-light-sensitive Chrimson-tdTomato and Chronos-GFP fibers, respectively, in a counter-balanced manner. The violet vertical bars above the traces indicate the photostimulations of either blue (λ = 473 nm) or red (λ = 638 nm) light. (I–M) Histograms of mean ± S.E.M. with circles denoting PPRs of individual neurons in SCx → LA and ACx → LA pathways. N.S., no significant difference, $^{*}p < 0.05$, $^{**}p < 0.01$, SCx → LA *vs*. ACx → LA, paired Student's *t*-test; N.S., no significant difference, $^{\#}p < 0.05$, $^{\#\#\#}p < 0.001$, ABB test *vs*. ABA renewal, unpaired Student's *t*-test. ABB test, n = 20 neurons from five mice; ABA renewal, n = 14 neurons from six mice.

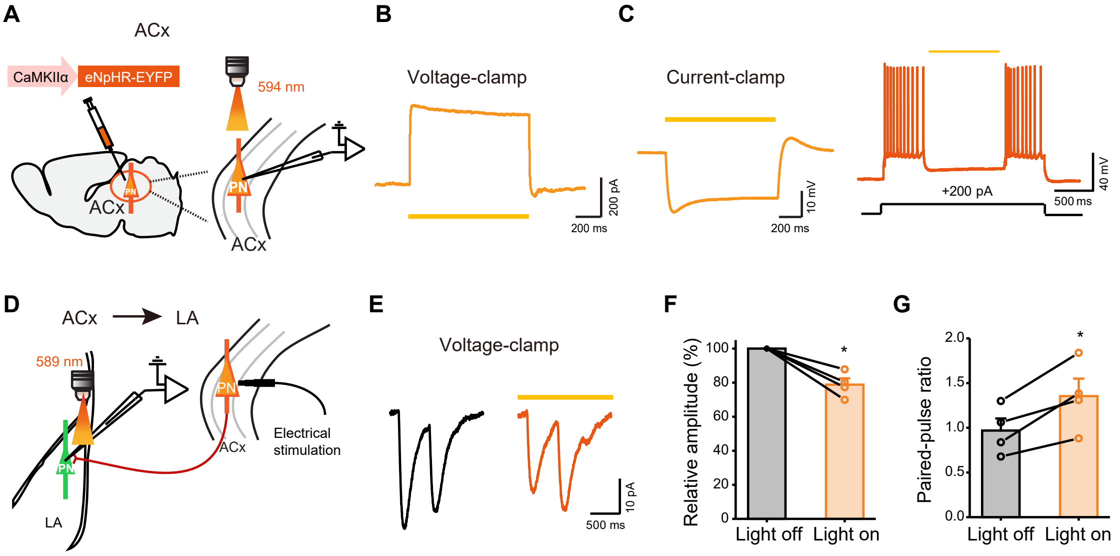

Supplementary Fig. 9. Characterization of functional expression of eNpHR-EYFP in ACx (A–C) and effects of eNpHR activation on the ACx → LA synaptic projection in LA (D–G).

(A) Experimental schemes to characterize the functional expression of eNPHR-EYFP.

(B and C) Representative traces of yellow light-evoked (1 s; continuous) outward current (B), membrane hyperpolarization and blockade of action potential firing recorded from eNpHR-EYFP-expressing neurons (C).

(D) Experimental schemes to characterize the effects of eNPHR action on the ACx → LA synaptic projection. The ACx was stimulated electrically with an electrode placed onto ACx, whereas eNpHR-expressing ACx fibers were stimulated with continuous yellow light (λ = 589 nm) in the LA. Synaptic responses were recorded in the principal neurons of the LA.

(E) Representative traces of EPSCs evoked by stimulation of the ACx-LA pathway with or without photostimulation of eNpHR on ACx fibers. The yellow horizontal bars above the trace indicates the yellow light photostimulation.

(F and G) Statistical results: $^{*}p < 0.05$, paired Student's *t*-test. n = 4 neurons from two mice.

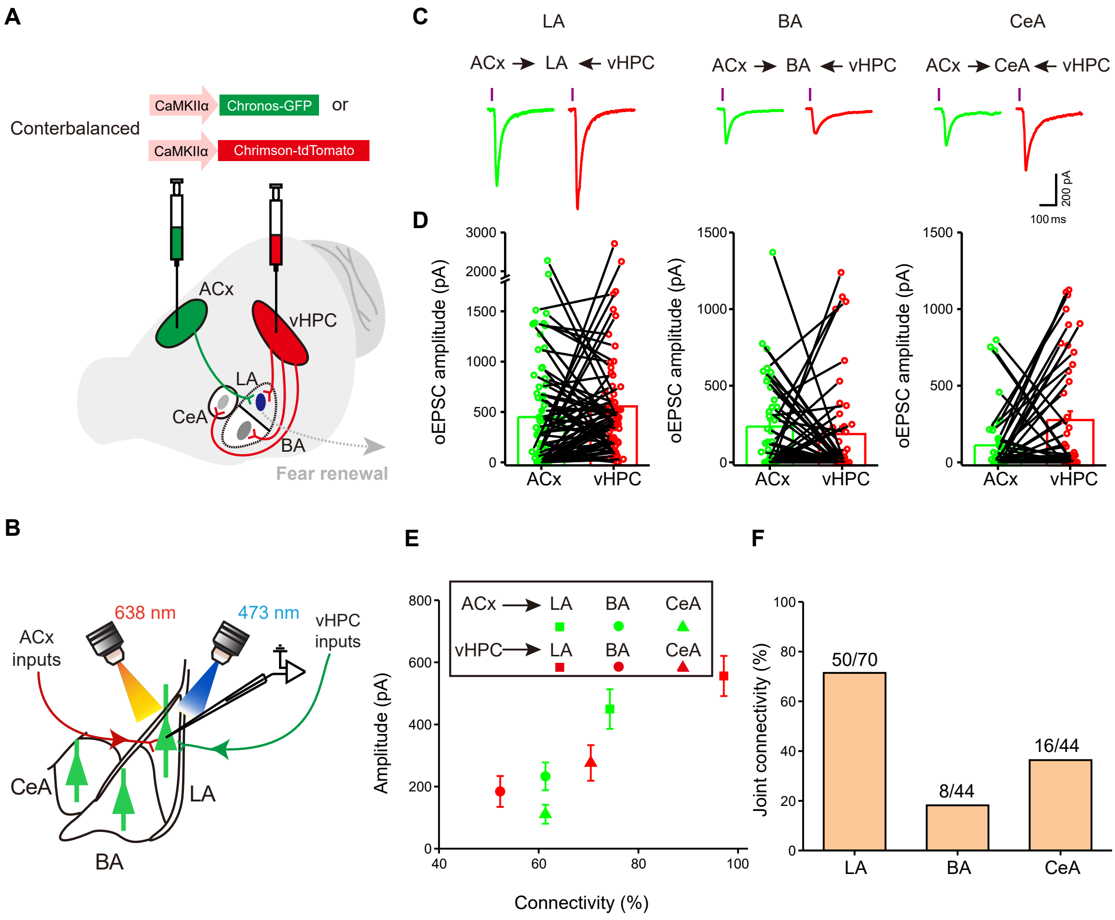

Supplementary Fig. 10. Electrophysiological characterization of amygdalar neurons receiving projections from ACx and vHPC.

(A) Schematics of AAV injections.

(B) Experimental schemes.

(C) Representative traces of oEPSCs recorded from the same LA, BA, or CeA neurons that received optical activation of the ACx or vHPC projections through photostimulation (violet vertical bars) of red- or blue-light-sensitive Chrimson-tdTomato and Chronos-GFP fibers, respectively, in a counter-balanced manner. The violet vertical bar above the trace indicates the photostimulation of either blue ($\lambda = 473$ nm) or red ($\lambda = 638$ nm) light.

(D) Histograms of mean ± S.E.M. with circles denoting oEPSC amplitudes of individual neurons in ACx → LA, vHPC → LA, ACx → BA, vHPC → BA, ACx → CeA, and vHPC → LA pathways. Solid lines connect the same neurons.

(E) Successful rate of optical stimulation of neurons in the LA, BA, and CeA subdivisions *via* the ACx and vHPC projections, and the mean oEPSC amplitudes.

(F) Joint connectivity based on the proportion of neurons that reacted to photostimulations of both ACx and vHPC inputs in the LA, BA, and CeA subdivisions. LA: n = 70 neurons from nine mice; BA: n = 44 neurons from nine mice; CeA: n = 44 neurons from nine mice.

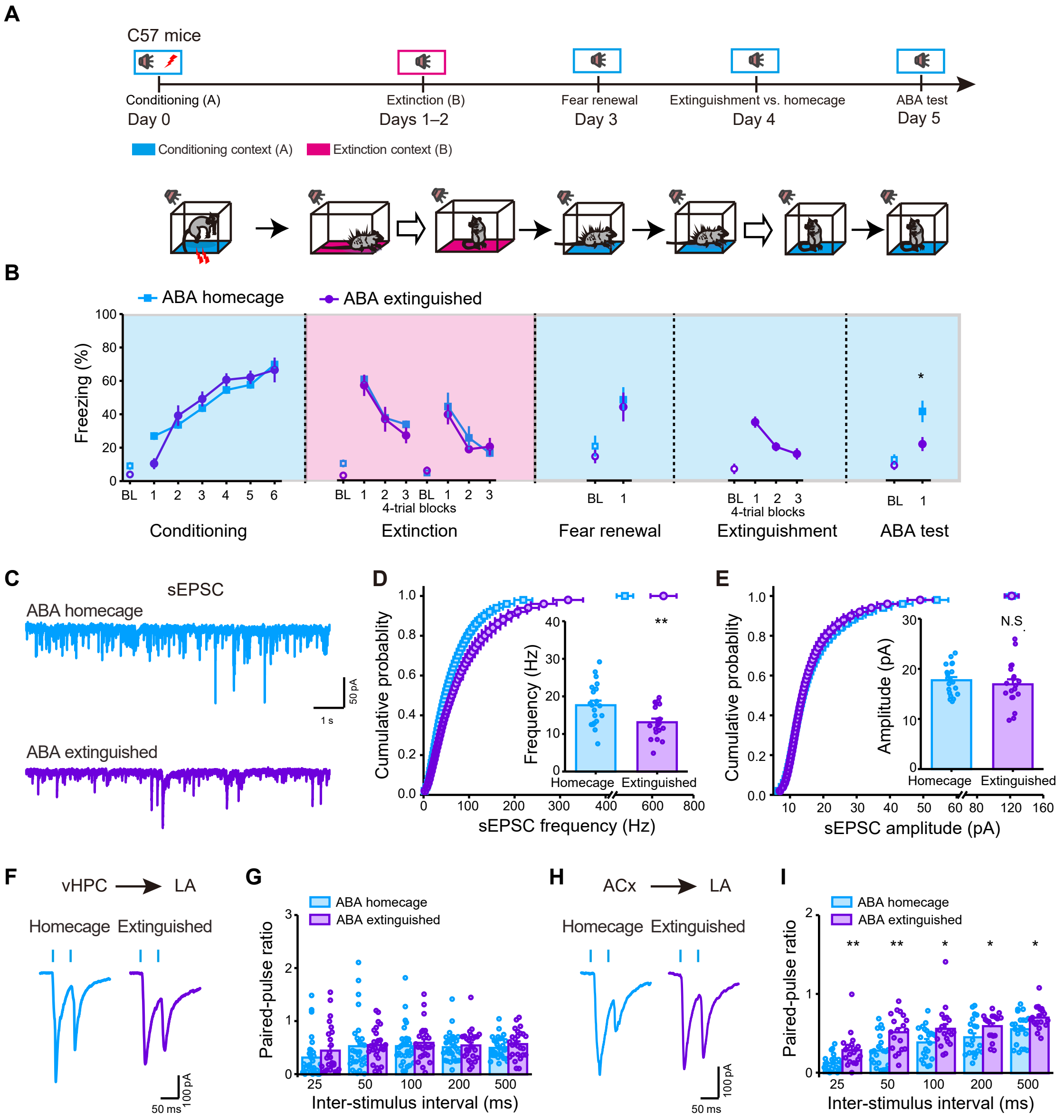

Supplementary Fig. 11. Effects of additional extinction training following fear renewal on synaptic adaptations of hippocampal and cortical inputs into LA.

(A and B) Behavioral characterization of additional extinction training following fear renewal. (A) Experimental schemes. (B) Freezing responses to the context only (baseline, BL) or CS. While the data points during conditioning represent the freezing period within individual CS presentations, those during extinction learning, fear renewal, and the subsequent extinguishment and memory test are averages of freezing responses to CS presentations of four consecutive trials. Statistics are as follows: two-way repeated measures ANOVA, main effect of behavior, conditioning, $F_{1,118} = 0.009$, $p = 0.923$; extinction learning, $F_{1,118} = 0.745$, $p = 0.390$; $^{*}p < 0.05$, unpaired Student's *t*-test. ABA homecage, n = 10, ABA extinguished, n = 10.

(C–E) Effects of additional extinction training following fear renewal on sEPSCs (as global synaptic inputs) in LA neurons. (C) Representative traces of sEPSCs in LA

neurons. (D and E) Cumulative distribution plots for sEPSC frequency (D) and amplitude (E). The inset shows values for individual neurons and the as summary data. N.S., no significant difference, $^{**}p < 0.01$, unpaired Student's *t*-test. ABA homecage, n = 21 neurons of three mice; ABA extinguished, n = 19 neurons of three mice.

(F–I) Effects of additional extinction training following fear renewal on paired-pulse ratios (PPRs) of oEPSCs at the vHPC → LA (F and G) and ACx → LA (H and I) projections. (F and H) Representative traces of oEPSCs at the vHPC → LA (F) or ACx → LA (H) synapses in the ABA homecage and ABA extinguished mouse groups induced by paired photostimulations (blue vertical bars) with a 50-ms interval. (G and I) Histograms of mean ± S.E.M. with circles denoting individual neurons. (G) vHPC → LA. Statistics: two-way repeated measures ANOVA, main effect of behavior, $F_{1,278} = 2.308$, $p = 0.130$. ABA homecage, n = 30 neurons from three mice; ABA extinguished, n = 26 neurons from three mice. (H) ACx → LA. Statistics: two-way repeated measures ANOVA, main effect of behavior, $F_{1,198} = 37.406$, $p < 0.001$; $^{*}p < 0.05$, $^{**}p < 0.01$, unpaired Student's *t*-test; ABA homecage, n = 21 neurons from three mice; ABA extinguished, n = 19 neurons from three mice.
